# Cell-Type Transcriptomes of the Multicellular Green Alga *Volvox carteri* Yield Insights into the Evolutionary Origins of Germ and Somatic Differentiation Programs

**DOI:** 10.1534/g3.117.300253

**Published:** 2017-12-05

**Authors:** Gavriel Y. Matt, James G. Umen

**Affiliations:** * Donald Danforth Plant Science Center, St. Louis, Missouri 63132; † Division of Biology & Biomedical Sciences, Washington University in St. Louis, Missouri 63110

**Keywords:** cell differentiation, multicellularity, green algae, evolution, carbon metabolism, stem cells, cooption, photosynthesis, flagella, basal bodies, senescence, extracellular matrix, secretory pathway

## Abstract

Germ–soma differentiation is a hallmark of complex multicellular organisms, yet its origins are not well understood. *Volvox carteri* is a simple multicellular green alga that has recently evolved a simple germ–soma dichotomy with only two cell-types: large germ cells called gonidia and small terminally differentiated somatic cells. Here, we provide a comprehensive characterization of the gonidial and somatic transcriptomes of *V. carteri* to uncover fundamental differences between the molecular and metabolic programming of these cell-types. We found extensive transcriptome differentiation between cell-types, with somatic cells expressing a more specialized program overrepresented in younger, lineage-specific genes, and gonidial cells expressing a more generalist program overrepresented in more ancient genes that shared striking overlap with stem cell-specific genes from animals and land plants. Directed analyses of different pathways revealed a strong dichotomy between cell-types with gonidial cells expressing growth-related genes and somatic cells expressing an altruistic metabolic program geared toward the assembly of flagella, which support organismal motility, and the conversion of storage carbon to sugars, which act as donors for production of extracellular matrix (ECM) glycoproteins whose secretion enables massive organismal expansion. *V. carteri* orthologs of diurnally controlled genes from *C. reinhardtii,* a single-celled relative, were analyzed for cell-type distribution and found to be strongly partitioned, with expression of dark-phase genes overrepresented in somatic cells and light-phase genes overrepresented in gonidial cells- a result that is consistent with cell-type programs in *V. carteri* arising by cooption of temporal regulons in a unicellular ancestor. Together, our findings reveal fundamental molecular, metabolic, and evolutionary mechanisms that underlie the origins of germ–soma differentiation in *V. carteri* and provide a template for understanding the acquisition of germ–soma differentiation in other multicellular lineages.

Germ–soma or reproductive division of labor is a hallmark of complex multicellular organisms such as plants and animals. In organisms with germ–soma division of labor, somatic cells forgo reproduction and become specialized for support functions, while germ cells or stem cells retain reproductive potential (Buss 1983, 1987; Denis and Lacroix 1993; Agata *et al.* 2006; Seydoux and Braun 2006; Johnson *et al.* 2011; Solana 2013). Although multicellularity without germ–soma division of labor has arisen repeatedly (*e.g.*, syncytial fungi and simple colonial algae), some degree of segregated reproductive potential arose at least once independently within multicellular taxa belonging to five major eukaryotic groups: plants (red algae, green algae, and land plants), Amoebozoa (cellular slime molds), Opisthokonts (animals and fungi), Alveolates (ciliates), and Heterokonts (brown algae) (Bonner 1998; Grosberg and Strathmann 2007; Herron *et al.* 2013). At least two selective advantages are thought to be associated with germ–soma separation. The first is conflict mitigation, which reduces intercellular competition for resources by restricting reproduction to a limited number of germ cells (Buss 1983, 1987; Michod 1997; Kerszberg and Wolpert 1998; Wolpert and Szathmáry 2002). The second advantage is the potential for increased functional specialization of somatic cells whose size, shape, organelle contents, and other attributes can be released from the constraints of undergoing periodic mitosis and cytokinesis (Wolpert 1990; Koufopanou and Bell 1993; Koufopanou 1994; Nedelcu and Michod 2004; Ispolatov *et al.* 2012; Woodland 2016). Indeed, the most complex multicellular taxa, including plants and animals, possess somatic cell-types that are terminally differentiated and, in some cases, completely incapable of further proliferation (*e.g.*, anucleate mammalian red blood cells and plant vascular cells). The genetic and developmental mechanisms controlling germ–soma differentiation in animals and land plants have been extensively investigated (Strome and Lehmann 2007; Dickinson and Grant-Downton 2009; Lesch and Page 2012; Schmidt *et al.* 2012, 2015; Strome and Updike 2015; Swartz and Wessel 2015), but the highly-derived body plans and ancient origins of these taxa make it challenging to infer the early evolutionary steps that generated their germ–soma dichotomies.

The multicellular green alga *Volvox carteri* is a member of a monophyletic group called the volvocine green algae, which includes multicellular species with complete germ–soma differentiation (*e.g.*, *V. carteri*), multicellular species lacking germ–soma differentiation (*e.g.*, *Gonium pectorale*), and unicellular species (*e.g.*, *Chlamydomonas reinhardtii*) (Nozaki *et al.* 2000; Kirk 2005; Nishii and Miller 2010; Herron 2016). Importantly, multicellularity and germ–soma differentiation arose relatively recently in volvocine algae (∼200 MYA) (Herron *et al.* 2009), making them attractive models for elucidating the origins of multicellular innovations (Kirk 1998, 2005; Nishii and Miller 2010; Umen and Olson 2012).

In its asexual phase, *V. carteri* possesses a simple spheroidal body plan with only two cell-types: ∼16 large aflagellate germ cells called gonidia that are positioned within the spheroid interior and ∼2000 small terminally differentiated somatic cells spaced evenly around the surface layer of the spheroid with flagella projecting outwards (Figure 1A). The majority of the adult spheroid volume is composed of clear secreted glycoprotein extracellular matrix (ECM) that maintains relative cell positioning and spheroid integrity (Hoops 1993; Hallmann and Kirk 2000; Kirk and Nishii 2001). Somatic cells provide phototactic motility to the spheroid through the coordinated beating of their flagella, and they secrete ECM that drives spheroid enlargement; however, somatic cells are terminally differentiated and eventually senesce and die. Gonidial cells serve a reproductive role by undergoing a period of cell growth followed by embryonic cleavage divisions and morphogenesis to produce a new generation of spheroids. Under optimal conditions, the entire vegetative life cycle of *V. carteri* can be synchronized under a 48 hr diurnal cycle (Kirk 1998, 2001; Kirk and Nishii 2001; Matt and Umen 2016) (Figure 1B).

**Figure 1:**
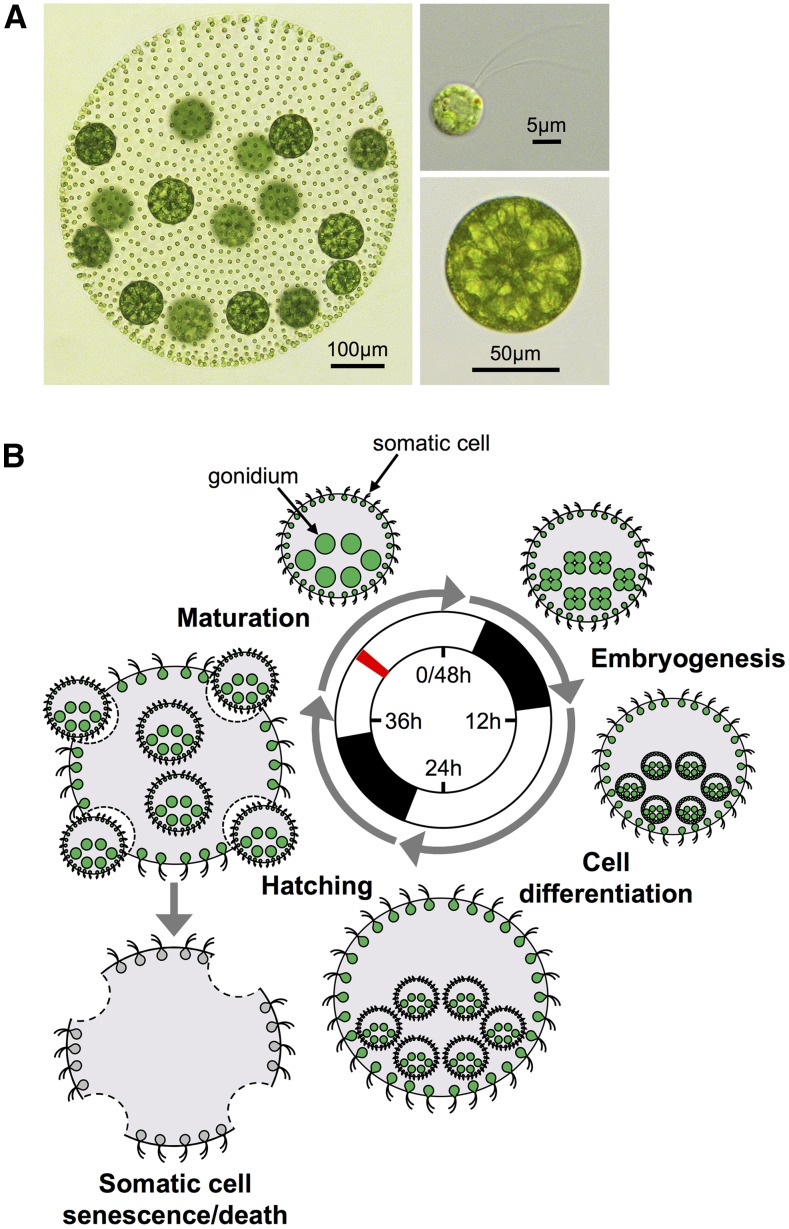
*V.* *carteri* cell-types and vegetative life cycle. (A) Micrographs of an intact adult spheroid with fully mature somatic and gonidial cells (left), isolated somatic cell (top right), and isolated gonidial cell (bottom right). (B) Diagram of the *V. carteri* vegetative (asexual) life cycle modified from Matt and Umen (2016). Center, 2 day diurnal regime used to synchronize *V. carteri* development with alternating 16 hr light (open bars) and 8 hr dark (closed bars) periods. Stages of the asexual life cycle are depicted proceeding clockwise from upper left (mature adult stage, black arrows indicate gonidial and somatic cell-types). Large green circles represent gonidial cells and small green circles with flagella represent somatic cells. Extracellular matrix is shaded gray. Gonidia within adult spheroids undergo cleavage and embryogenesis to form embryos with large gonidial precursors and small somatic precursors. Embryos undergo cytodifferentiation, where somatic precursors elongate their flagella and gonidial precursors grow in size, giving rise to juvenile spheroids. Juvenile spheroids eventually hatch from their mother spheroid whose somatic cells undergo senescence and death. Mature adult stage spheroids were used for cell-type separation and RNA extraction. Red line indicates the period in the diurnal cycle when spheroids were harvested.

Germ–soma differentiation is under genetic control in *V. carteri* , as evidenced by developmental mutants such as *regA*^−^, whose somatic cells are formed initially but then redifferentiate into gonidial cells (Huskey and Griffin 1979; Kirk *et al.* 1999), and *lag*^−^ mutants, whose presumptive gonidial cells transiently differentiate as somatic cells but then acquire a gonidial identity (Kirk 1988, 1998, 2001). *regA* has been cloned and encodes a nuclear-localized putative transcription factor that is expressed only in somatic cells, where it represses gonidial cell fate (Kirk *et al.* 1999). It has been hypothesized that cell-type-specific genes in *V. carteri*, including targets of RegA, may have arisen through cooption of temporally or environmentally controlled genes from a unicellular ancestor that came under cell-type control in *V. carteri* (Nedelcu and Michod 2004, 2006; Nedelcu 2009), but this idea has not been tested on a genomic scale.

Previous studies have identified a few dozen cell-type-specific genes in *V. carteri* using either differential hybridization or quantitative RT-PCR (Tam and Kirk 1991; Nematollahi *et al.* 2006; Kianianmomeni and Hallmann 2015). Notably, Tam and Kirk (1991) identified 19 gonidial-specific genes, and subsequent annotation of these genes revealed that 13/19 of the encoded proteins were involved in photosynthesis and other chloroplast-related functions (Choi *et al.* 1996; Meissner *et al.* 1999). This result led to the proposal that germ–soma dichotomy in *V. carteri* is achieved by targeted suppssion of chloroplast biogenesis and photosynthesis in somatic cells (Meissner *et al.* 1999). However, the suppression of photosynthetic-related gene expression as the possible basis for germ–soma differentiation has not been explored further.

Here, we used deep transcriptome sequencing of highly purified gonidial and somatic cells of *V. carteri* to characterize their respective differentiation programs. Using intra- and interspecific genomic comparisons we found widespread differences in the compositions and phylogenetic origins of the two transcriptomes, which highlight fundamental differences between germ and somatic cell-types. Moreover, the cell-type transcriptomes in *V. carteri* could be correlated with temporally regulated diurnal regulons in *C. reinhardtii*, lending support to the temporal–spatial cooption hypothesis for the origins of *V. carteri* cell-types. Although our data did not support the photosynthetic suppression hypothesis for somatic differentiation, we did find strong evidence for extensive coordinated cellular and metabolic differentiation between cell-types, with anabolic pathways expressed preferentially in gonidial cells and a catabolic program in somatic cells that appeared specialized for the conversion of carbon stores into sugars that are the major constituent of the ECM glycoproteins.

## Materials and Methods

### Strains and culture conditions

*V. carteri* f. *nagariensis* female strain Eve (HK10), which has been maintained by subculturing since first isolated (Starr 1970), was obtained from Stephen Miller (University of Maryland, Baltimore County) and used for all experiments. Cultures were maintained in Standard Volvox Medium (SVM) in 30 ml glass tubes under fluorescent white light and synchronized under a 16 hr light:8 hr dark cycle at 32° (Tam and Kirk 1991). For cell-type separation and RNA preparation, cultures were grown in 500 ml flasks at 32° in temperature-controlled water baths aerated by bubbling air, and synchronized under a 16 hr light:8 hr dark cycle with illumination from LED lights [250 µEm^−2^s^−1^ total, 1:1 fluence ratio of red (625 nm) and blue (465 nm)].

### Separation of cell-types

Separation of cell-types was based on a protocol from Tam and Kirk (1991), but modified to reduce processing time and to obtain purer cell-type preparations. Replicate cultures were used to prepare each cell-type. To avoid delays in sample processing only one cell-type was prepared from a given culture. For each of four cell-type preparations (two gonidial and two somatic), 10 flasks containing 350 ml SVM were inoculated with 15–20 manually selected cleavage-stage spheroids and cultures were grown for two generations (90 hr) to a density of 3000–4000 spheroids per flask. Hatched adult spheroids were harvested prior to the initiation of gonidial cleavage (hour 42 in the 48 hr life cycle, which is 6 hr into the second light period and ∼6 hr prior to the initiation of embryonic cleavage, Figure 1B) by collection onto a 30 µM nylon mesh filter (catalog# 03-30/18; SEFAR), and then transferred to a 40 ml Kimble Kontes Dounce Tissue Grinder and suspended in 40 ml SVM.

For gonidial cell purification, spheroids were disrupted by three to four passages of a loose-fitting (A) pestle, and the disrupted spheroid mixture was split into two 50 ml conical tubes and diluted 1:1 with SVM. Percoll (Sigma) was added to each tube to a final concentration of 7% (v/v), and tubes were centrifuged at 100 × *g* for 2 min in an Eppendorf 5810 centrifuge at room temperature. Supernatants from each tube were discarded and gonidial cell pellets were resuspended in 20 ml SVM, after which cell suspensions were combined and centrifuged at 100 × *g* for 1 min. Gonidial cells were washed again with SVM using the same centrifugation conditions, resuspended in 10 ml SVM, and then examined visually for purity using a dissecting microscope. Purified cells were transferred to a 15 ml tube and then immediately processed for RNA isolation (see below).

For somatic cell purification, harvested spheroids were disrupted by five passages of a tight-fitting (B) pestle, and the disrupted spheroid mixture was split into two 50 ml conical tubes and diluted 1:1 with SVM. Percoll was added to each tube to a final concentration of 7% (v/v) and tubes were centrifuged at 200 × *g* for 5 min. The somatic cell sheets at the top of the gradient in each tube were pipetted into 200 ml SVM and then split into four 50 ml conical tubes. Somatic cell sheets were pelleted at 3220 × *g* for 3 min and resuspended in 5 ml of SVM, after which they were combined into a single 50 ml conical tube and diluted 1:1 with fresh SVM. Cells were recentrifuged using the above conditions, resuspended in 10 ml SVM, and then examined visually for purity using a dissecting microscope. Purified cells were transferred to a 15 ml tube and then immediately processed for RNA isolation.

### RNA isolation

Cells were pelleted and then resuspended in 5 ml Buffer C (2% SDS in Tris-Buffered Saline) and 5 ml of Trizol (Invitrogen), and then immediately flash frozen in liquid nitrogen and stored at −80°. Lysates were thawed, transferred to a 16 × 100 mm Covaris glass tube (SKU: 500012), and homogenized in a Covaris S220 ultrasonicator at 4° with the following settings: first treatment: Peak Power = 300, Duty Factor = 20, Cycles/Burst = 500, Temperature = 5°, and Time = 200 sec; and second treatment: Peak Power = 250, Duty Factor = 20, Cycles/Burst = 500, Temperature = 5°, and Time = 200 sec. Tubes with lysates were inverted to mix in between the first and second treatments. Lysates were centrifuged at 3000 × *g* for 10 min at room temperature and supernatants were extracted with 3 ml chloroform in a MaXtract High Density tube (QIAGEN). The aqueous phase was transferred to a 15 ml conical tube, and RNA was precipitated by adding 5 ml of isopropanol and incubating at room temperature for 10 min. RNA was pelleted by centrifugation at 3000 × *g* for 10 min and then washed with 75% ethanol. DNase digestion and purification of RNA were done using an RNeasy Mini Kit (QIAGEN) according to the manufacturer’s instructions. RNA concentration was determined by measuring absorbance at 260 nm using a nanophotometer (Implen). RNA quality was evaluated by agarose gel electrophoresis and using an Agilent 2100 Bioanalyzer.

### Library preparation and deep sequencing

Two biological replicate samples of gonidial and somatic RNA were submitted to the Genome Technology Access Center in the Department of Genetics at Washington University School of Medicine for Illumina-based polyA library preparation and sequencing. oligo-dT-selected RNA was fragmented to an average size of ∼200 nt and reverse transcribed to produce double-stranded cDNA using random hexamers. cDNA ends were end repaired, 3′ dA-tailed, and then ligated to Illumina sequencing adapters. Adaptor-ligated fragments were amplified for 12 cycles using indexed primers and sequenced on an Illumina HiSeq-2500 using single end reads extending 50 bases.

### Mapping sequencing reads and classifying cell-type-specific gene expression

**Figure 2:**
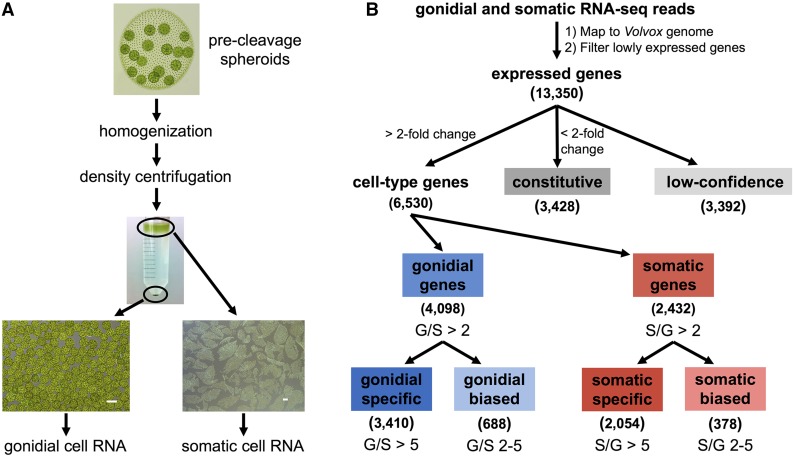
Cell-type sample preparation and gene expression classification. (A) Precleavage spheroids (top micrograph) were disrupted to separate cells types, and then subjected to differential density centrifugation (middle micrograph) to produce separate pure populations of somatic cell sheets on top of the gradient and gonidia in the pellet. Micrographs of separated cell-types at the bottom show sample purity (scale bars = 100 µm). (B) Bioinformatic workflow for classifying cell-type gene expression based on gonidial/somatic expression ratios (see *Materials and Methods* for details). Numbers in parentheses indicate number of genes in each expression category. G, gonidial expression; S, somatic expression.

Quality of gonidial and somatic reads was evaluated using FastQC (Andrews 2010). Reads were processed using the Trimmomatic tool (Bolger *et al.* 2014) by removing adaptor sequences, low-quality bases (quality score < 3) from the 5′ and 3′ ends of reads, and the 3′ ends of reads after the average quality score in a sliding four-base window dropped below 15. Trimmed reads shorter than 25 nt were discarded. Processed reads were aligned to the *V. carteri* V2 genome assembly available on Phytozome (https://phytozome.jgi.doe.gov) (Prochnik *et al.* 2010; Goodstein *et al.* 2012) using STAR (Dobin *et al.* 2013), with the maximum number of mapped loci set to 10 and a maximum mismatch ratio of 0.06 (Supplemental Material, Table S1 in File S1). For assigning mapped reads to gene models, a merged set of protein-coding gene models was generated by combining all 14,247 *V. carteri* version 2.1 gene models currently available on Phytozome (Prochnik *et al.* 2010; Goodstein *et al.* 2012) with 1761 nonoverlapping (<20% sequence overlap) version 2.0 Phytozome protein-coding gene models (Prochnik *et al.* 2010; Goodstein *et al.* 2012) to generate a final set of 16,008 unique protein-coding gene models. Uniquely mapped reads were assigned to the merged set of gene models using HTSeq (Anders *et al.* 2014). Genes with no expression in any sample were removed from the analysis. Raw read counts for each gene in each sample were used as input for differential expression analysis using DESeq2 (Love *et al.* 2014). Following the removal of the upper 0.3% quantile of read counts in each sample, the total read count in each sample was divided by 10 million to yield size factors that were used to normalize expression estimates in each sample in DESeq2 (Bullard *et al.* 2010). Finally, genes in the bottom 10th quantile of normalized expression were removed from the analysis, as this cutoff was shown to maximize the number of significantly differentially expressed genes by minimizing the impact of multiple testing. These filtering steps yielded a set of expressed genes in the transcriptome that were then classified based on their cell-type expression patterns. Genes were considered to be gonidial or somatic genes if they exhibited at least a twofold difference in expression between cell-types with a false discovery rate (FDR) < 0.05. Gonidial and somatic genes were further subclassified based on expression ratio, with those exhibiting a two- to fivefold cell-type expression ratio designated as cell-type-biased, and those having greater than a fivefold expression ratio designated as cell-type-specific (Figure 2B). The remaining genes, all of which had an FDR > 0.05, were classified as “constitutive” if their expression ratio was <2 or “low confidence” if their expression ratio was >2. The low-confidence genes generally had cell-type expression ratios clustered around the twofold threshold but lacked statistical support for classification as cell-type-biased (Figure 2B and Figure S2 in File S1). We also calculated RPKM (reads per kilobase per million mapped reads) normalized expression values (Mortazavi *et al.* 2008) for all *V. carteri* genes after adding 0.1 read counts to each gene in order to eliminate zeros in expression ratio calculations. Expression ratios derived from DESeq2 and RPKM normalization were highly correlated (*r*^2^ = 0.995) (Figure S9 in File S1), and RPKM expression values were used for all further analyses and reported in Datasets S1, S2, S3, S4, S5, S6, S7, S8, S9 and S10.

### Identifying V. carteri orthologs and coorthologs of C. reinhardtii genes

BLASTP (Altschul *et al.* 1990), with a BLOSUM80 scoring matrix and masking of low complexity and simple repeat sequences, was used to query *V. carteri* predicted proteins (derived from the merged set of gene models described above; 16,008 proteins) against all *C. reinhardtii* v5.5-predicted proteins available on Phytozome (17,741 proteins) (Merchant *et al.* 2007; Goodstein *et al.* 2012). Alignments were processed using the Smith–Waterman algorithm (Smith and Waterman 1981). Based on manual inspection of alignments, an E-value threshold of 1e−10 and a query coverage of >50% was chosen as a cutoff for homology assignment. *V. carteri* orthologs of *C. reinhardtii* proteins were assigned based on a mutual best hit criterion (Moreno-Hagelsieb and Latimer 2008): protein A in *V. carteri* (VcaA) was considered to be an ortholog of protein A in *C. reinhardtii* (CreA) if the alignment score between VcaA and CreA was higher than the alignment score between any another *V. carteri* protein and CreA. *V. carteri* coorthologs (or in-paralogs) were defined as follows: if VcaA and CreA are orthologs, then VcaB is a coortholog of CreA if CreA is the best hit for VcaB and if the VcaB-VcaA alignment score is higher than the VcaA-CreA alignment score. Using these criteria, we identified 9184 mutual best hit relationships between *V. carteri* and *C. reinhardtii* proteins, and 340 additional *V. carteri* proteins that were assigned as co-orthologs. We used these orthology relationships to annotate *V. carteri* orthologs and coorthologs of *C. reinhardtii* genes related to flagella, basal bodies, photosynthesis, central carbon metabolism, glycosylation, and autophagy, which are reported in Datasets S4, S5, S6, S8, and S10.

### Functional classification of protein-coding genes

To assign MapMan terms to *V. carteri* protein-coding genes, we utilized classification terms already assigned to *C. reinhardtii* version 5.3.1 genes (http://mapman.gabipd.org/) (Thimm *et al.* 2004). v5.3.1 genes were first converted to version 5.5 *C. reinhardtii* IDs using a conversion table available on Phytozome (C. reinhardtiiTranscriptNameConversionBetweenReleases.Mch12b.txt.gz) (Merchant *et al.* 2007; Goodstein *et al.* 2012). *C. reinhardtii* annotation terms were then assigned to *V. carteri* orthologs as defined above. Statistical enrichment tests for MapMan terms in *V. carteri* cell-types were performed using all expressed *V. carteri* genes (defined above in *Mapping sequencing reads and classifying cell-type-specific gene expression*) as a background model. For each MapMan annotation level, we used the two-sided Fisher’s exact test with an odds ratio > 1 and an FDR < 0.05 as criteria for cell-type enrichment. Gene ontology (GO) terms were assigned using Blast2GO (Götz *et al.* 2008) with default settings, and with input derived from a BLASTP search of the predicted *V. carteri* proteome queried against the NCBI nonredundant database with a significance threshold of 1e−3. Additional GO terms were identified in Blast2GO using InterProScan (Quevillon *et al.* 2005), ANNEX (Myhre *et al.* 2006), and GO-slim (Camon *et al.* 2004) options. PFAM domain assignments to *V. carteri* proteins were made using the InterProScan output from Blast2GO.

### Phylostratigraphic analysis of cell-type-specific genes

The phylostratigraphy approach was based on a previously reported method (Domazet-Loso *et al.* 2007). We first assembled a database of proteomes from species representing different phylostrata or taxonomic categories (*V. carteri* , Volvocaceae, Volvocine algae, Chlorophyte green algae, Viridiplantae, Eukaryotes, and Cellular organisms) (Table S5). The *V. carteri* proteome was compared to all proteomes using BLASTP with a BLOSUM45 substitution-scoring matrix designed for comparing distantly related protein sequences (Henikoff and Henikoff 1992), with filtering of low complexity and simple repeat sequences. Based on manual inspection of alignments, an E-value threshold of <1e−20 and a query coverage >50% were chosen as similarity cutoffs for assigning homology. A phylostratum assignment for each *V. carteri* protein was made based on the most distant phylostratum in which at least one homolog was found. Enrichment for cell-type expression was tested using the two-sided Fisher’s exact test with all expressed *V. carteri* genes as a background, with significance at an odds ratio > 1 and an FDR < 0.05.

### Orthology group analysis of V. carteri genes, animal (metazoan) stem cell genes, and land plant (embryophyte) stem cell genes

Metazoan pluripotent stem cell genes have previously been classified into 180 OrthoMCL orthology groups (IDs) (Alié *et al.* 2015). To define a core set of land plant pluripotent stem cell genes, we used a published dataset that identified genes upregulated in the shoot apical meristem of the angiosperm *Zea mays* and genes upregulated in the gametophore apical cells of the moss *Physcomitrella patens* (Frank and Scanlon 2015). Protein sequences of the genes upregulated in the shoot apical meristem of *Z. mays* were acquired from a set of *Z. mays* version 2 predicted protein sequences (https://www.maizegdb.org/gene_center/gene#gm_downloads) (Andorf *et al.* 2016), and protein sequences of the genes upregulated in the gametophore apical cells of *P. patens* were acquired from a set of *P. patens* version 1.6 predicted protein sequences (https://www.cosmoss.org/physcome_project/wiki/Genome_Annotation/V1.6#GFF3) (Zimmer *et al.* 2013). We assigned these protein sequences to OrthoMCL database orthology groups (http://www.orthomcl.org) (Chen *et al.* 2006; Fischer *et al.* 2011), and then defined the intersection between the orthology groups containing maize meristem genes and the orthology groups containing *Physcomitrella* apical cell genes to be the set of land plant (embryophyte) stem cell orthology groups. All *V. carteri* proteins in the merged proteome were queried against the OrthoMCL database in a similar fashion as above, which allowed us to assign 11,636 *V. carteri* genes to 8356 unique orthology groups (IDs). Genes from each *V. carteri* orthology group were then queried for their expression classifications [gonidial, somatic, and not cell-type-regulated (constitutive + low confidence)]. If all *V. carteri* genes in an orthology group had the same expression classification, then the orthology group ID was assigned to that *V. carteri* expression classification. Orthology groups containing only genes with no or low expression in the transcriptome (defined in *Mapping sequencing reads and classifying cell-type-specific gene expression*), or orthology groups containing genes belonging to >1 expression classification, were removed from the analysis. The merged set of *V. carteri* OrthoMCL IDs associated with each *V. carteri* expression classification was used as a background for testing enrichment/deenrichment of different subsets using a two-sided Fisher’s exact test. Enrichment was defined as an odds ratio > 1 and an FDR < 0.05, while deenrichment was defined as an odds ratio < 1 and an FDR < 0.05.

### Phylogenetic analysis and expression quantification of the light harvesting complex (LHC) genes

*V. carteri* LHC proteins were identified using BLASTP searches (Altschul *et al.* 1990) with annotated LHCII, LHCI, and LHCSR proteins from *C. reinhardtii* (Zones *et al.* 2015). All alignments and tree building were done using MEGA7 (Kumar *et al.* 2016). Protein alignments were done using MUSCLE (Edgar 2004) within MEGA. A Jones–Taylor–Thornton amino acid substitution model with a γ parameter of two was used to estimate a neighbor joining tree with 1000 bootstrap replications. Due to the high sequence similarity between paralogs belonging to LHCII clade 1 (19 members) and LHCII clade 4 (seven members), expression of individual members within these two clades could not be assigned. Instead, each clade was treated as a single gene by remapping sequence reads to the *V. carteri* genome, with up to 19 matches allowed for clade 1 and up to seven matches allowed for clade 4, and allowing for no base mismatches. Mapped reads that intersected with clade 1 genes and clade 4 genes were identified using SAMtools (Li *et al.* 2009) and BEDTools (Quinlan and Hall 2010). The total read counts were calculated by counting each read mapping to any of the clade 1 genes or to any of the clade 4 genes as one read to the respective clade. The mean transcript length for LHCII genes from clade 1 or from clade 4 was used as a basis to estimate RPKMs for each clade.

### Annotation of central carbon metabolism in V. carteri

Metabolic pathways shown in Figure 7 were based on the following references: Calvin cycle (http://www.biocyc.org/CHLAMY/; Serrato *et al.* 2009), tricarboxylic acid (TCA) cycle (http://www.biocyc.org/CHLAMY/), amino acid metabolism (Vallon and Spalding 2009; Fontaine *et al.* 2012; De La Torre *et al.* 2014; Plancke *et al.* 2014), starch metabolism (Dauvillée *et al.* 2006; Ball and Deschamps 2009; Johnson and Alric 2013), fatty acid metabolism (Goepfert and Poirier 2007; Li-Beisson *et al.* 2015), glycolysis/gluconeogenesis (Klein 1986; Johnson and Alric 2013), glyoxylate cycle (Spalding 2009), and nucleotide sugar metabolism (http://www.genome.jp/kegg/pathway.html; Seifert 2004).

### Annotation of nucleotide sugar metabolism enzymes in V. carteri

We annotated biosynthetic pathways for nucleotide sugars that are the major glycosylation donors for cell wall/ECM glycoproteins in volvocine algae: UDP-arabinose, GDP-mannose, UDP-galactose/GDP-galactose, UDP-xylose, and UDP-glucose (Sumper and Hallmann 1998; Hallmann 2003; Harris 2009). *V. carteri* and *C. reinhardtii* genes predicted to encode enzymes involved in the biosynthesis of these nucleotide sugars were obtained from the KEGG website (http://www.genome.jp/kegg-bin/show_pathway?map=map00520&show_description=show) (Kanehisa *et al.* 2016). Downloaded *V. carteri* v1 gene models from KEGG were converted to version 2.1/2.0 gene models using BLASTN searching for correspondence (Altschul *et al.* 1990). *C. reinhardtii* v3 gene models from KEGG were converted to version 5.5. gene models using the conversion table described above followed by manual curation. *V. carteri* orthologs of *C. reinhardtii* genes were identified as described above. Putative UDP-galactopyranose mutases were identified in *V. carteri* by identifying orthologs of a *C. reinhardtii* UDP-galactopyranose mutase that was previously identified (Beverley *et al.* 2005) and identifying other *V. carteri* genes with the same EC annotation (5.4.99.9, UDP-galactopyranose mutase) in Phytozome. The full set of *V. carteri* genes predicted to be involved in nucleotide sugar metabolism is reported in Dataset S7.

### Annotation of glycosyltransferases in V. carteri

We identified glycosyltransferases that are involved in either O-glycosylation or modification of O-linked glycans, and whose substrates are the sugars found on *V. carteri* ECM glycoproteins (see above). Putative *V. carteri* glycosyltransferases were identified through a mutual best-hit approach as follows. Protein sequences of arabinosyltransferases and galactosyltransferases from *Arabidopsis thaliana* (Showalter and Basu 2016), mannosyltransferases from *Saccharomyces cerevisiae* (PMT1, AAA02928; KRE2, CAA44516; KTR6/MNN6, NP_015272; and MNN1, AAA53676), xylosyltransferases from *Homo sapiens* and *Phaseolus vulgaris* (GXYLT1, NP_001093120; C3ORF21, NP_001294998; TMEM5, NP_001265166; ZOX1, AAD51778; and XYLT1, NP_071449), and glucosyltransferases from *Drosophila melanogaster* and *Ph. lunatus* (Rumi, NP_001262849 and ZOG1, AAD04166) were queried against the *V. carteri* merged proteome using BLASTP (Altschul *et al.* 1990) (E-value cutoff = 1e−3) to identify candidate *V. carteri* homologs. Using this approach, we identified candidate *V. carteri* homologs of *A. thaliana* arabinosyltransferases and galactosyltransferases, *S. cerevisiae* mannosyltransferases, and *D. melanogaster* glucosyltransferases. The protein sequences of these candidate *V. carteri* homologs were then reciprocally queried against the predicted proteomes of the originally queried species [*A. thaliana* TAIR10 (https://phytozome.jgi.doe.gov) (Goodstein *et al.* 2012; Lamesch *et al.* 2012); *S. cerevisiae* S288C (http://www.yeastgenome.org/blast-sgd, last updated January 13, 2015) (Engel *et al.* 2014); and *D. melanogaster* (http://flybase.org/blast/, DmeI release 6.15) (Gramates *et al.* 2017)], using BLASTP to verify their similarity to the original enzyme classification and rule out better-scoring classifications. Candidate paralogs of the InvC protein in *V. carteri* (BAH03159), which encodes a LARGE glycosyltransferase that is required for extracellular vesicle expansion (Ueki and Nishii 2008), were also identified using BLASTP. Protein sequences of candidate InvC paralogs were then queried against the predicted proteome of *Mus musculus* GRCm38.p5 (https://www.ncbi.nlm.nih.gov/genome?term=mus%20musculus) (Church *et al.* 2009), using BLASTP to validate their homology to LARGE glycosyltransferases. The full set of *V. carteri* predicted glycosyltransferases is reported in Dataset S8.

### Cell-type expression patterns of paralogous genes encoding central carbon metabolism enzymes

A number of paralogous gene groups encoding isoforms of the same central carbon metabolism enzyme (*e.g.*, Rubisco small subunit) were considered functionally similar, unless there was evidence available about differential isoform localization or differential function in algae or land plants. Expression estimates of functionally similar paralogs were treated additively, based on the rationale that summed expression provides a better estimate of relative activity in each cell-type, especially in cases where one isoform is clearly dominant (Figure 7 and Datasets S6 and S7). These summed cell-type RPKM levels were then used to calculate cell-type expression ratios for each gene group. Assignment of each gene group into a cell-type expression classification was based on the same criteria described for single genes in *Mapping sequencing reads and classifying cell-type-specific gene expression*, but no confidence statistics were calculated for the expression ratios of summed paralogs.

### Predicting subcellular localizations of V. carteri proteins

Predictions for subcellular localizations of all *V. carteri* predicted proteins from the merged set of gene models described above were done using PredAlgo (https://giavap-genomes.ibpc.fr/cgi-bin/predalgodb.perl?page=main) (Tardif *et al.* 2012). Enrichment testing (Figure S6 in File S1) was done using a two-sided Fisher’s exact test with FDR < 0.05, with the subcellular localization distribution for all *V. carteri* proteins in the expressed transcriptome used as a background model.

### Correspondence between V. carteri cell-type-regulated genes and C. reinhardtii diurnal clusters

We identified *V. carteri* orthologs of *C. reinhardtii* genes found in each of 18 diurnal expression clusters and an unclustered group (Zones *et al.* 2015) (coorthologs were excluded from the analysis). *V. carteri* orthologs with either low expression or those within the low-confidence expression classification were removed from the analysis. The cell-type expression pattern classifications for *V. carteri* orthologs derived from each *C. reinhardtii* diurnal cluster were then tested for relative enrichment using a two-sided Fisher’s exact test with an FDR < 0.05, with the distribution of cell-type expression pattern classifications for all *V. carteri* orthologs in the analysis used as a background distribution. A reciprocal analysis was also performed by first merging the *C. reinhardtii* diurnal clusters into four major superclusters: light-phase genes from clusters 1–8, light–dark transition-phase genes from diurnal clusters 9–11, dark-phase genes from diurnal clusters 12–18, and a group of unclustered genes. The supercluster classifications for *C. reinhardtii* orthologs of *V. carteri* gonidial genes, somatic genes, and constitutive genes were then tested for relative enrichment using a two-sided Fisher’s exact test with an FDR < 0.05, with the distribution of supercluster classifications for all *C. reinhardtii* orthologs of all expressed *V. carteri* genes used as a background distribution. When testing the effect of flagella genes on the distributions of supercluster classifications, flagella genes (Dataset S4) were removed from the analysis and significance testing was redone.

### Data availability

Wild-type *V. carteri* f. *nagariensis* strain Eve (HK10) can be obtained from UTEX (http://utex.org/) or NIES (http://mcc.nies.go.jp/), and our current subclone is available upon request. Names and descriptions of supplemental tables and datasets can be found in the supplemental material. Primary data are available at the NCBI Gene Expression Omnibus repository under accession number GSE104835. *V. carteri* locus ID numbers for genes described in this study are listed in the appropriate supplemental datasets and can be accessed from Phytozome (http://phytozome.jgi.doe.gov).

## Results

### Identification of germ- and somatic-specific genes

To characterize the germ and somatic transcriptomes of *V. carteri*, we purified gonidial and somatic cells from hatched, precleavage adult spheroids (Figure 1B and Figure 2A). This life cycle stage was chosen for three reasons: (1) adult spheroids have fully differentiated germ and somatic cell-types; (2) since gonidia are not dividing the cell-type comparisons are not complicated by gene expression programs associated with cell division and embryogenesis; and (3) the two cell-types have the largest difference in size and are easiest to separate into pure populations (Figure 2A).

RNA was prepared from two biological replicates of each cell-type and processed for high-throughput Illumina-based sequencing. First, 15–17 million single-end reads were generated from each RNA-seq library, with 80–90% of reads mapping uniquely to version 2 of the *V. carteri* genome assembly (Prochnik *et al.* 2010) (Table S1 in File S1). A nonoverlapping set of 16,008 gene models comprised of version 2.0 and version 2.1 predictions from Phytozome was used for quantitative analysis of gene expression (*Materials and Methods*). After filtering out poorly expressed genes we obtained a set of 13,350 expressed genes (∼83% of predicted genes), which were subjected to analyses for differential expression between cell-types. Replicate samples for each cell-type had highly correlated expression profiles, while comparisons between cell-types indicated a large number of differentially expressed genes (Figures S1 and S2 in File S1). We classified genes into different categories based on their cell-type expression patterns (*Materials and Methods*) (Figure 2B). Genes that had at least a twofold difference in expression between cell-types were classified as either gonidial or somatic genes based on whether they had higher expression levels in gonidial cells or somatic cells, respectively. Gonidial genes that had between a two- and fivefold gonidial/somatic expression ratio were considered gonidial-biased (688 genes, ∼5% of the transcriptome), and those with greater than a fivefold gonidial/somatic expression ratio were considered gonidial-specific (3410 genes, ∼25%). Somatic genes were classified in a similar manner into somatic-biased (378 genes, ∼3%) and somatic-specific subsets (2054 genes, ∼15%). Genes that had less than a twofold difference in expression between cell-types were classified as constitutive (3428 genes, ∼25%). An additional 3392 genes (∼25%) had cell-type expression ratios close to the twofold cutoff for classification as cell-type genes, but lacked statistical support for differential expression and were classified as low confidence (Figure 2B and Figure S2 in File S1).

The purity of cell-type-specific RNA preparations was validated by examining the expression pattern of a known somatic-specific gene, *regA* (Kirk *et al.* 1999), whose average transcript level in somatic cells (6.594 RPKM) was nearly 1000-fold higher than in gonidial cells, where it was at or near the detection limit (0.007 RPKM) (Datasets S1 and S2). Moreover, we identified numerous other genes whose expression was highly specific (>1000-fold expression ratio) for one or the other cell-type (Table S2 in File S1), a finding that rules out the presence of significant cross-contamination between cell-type preparations. Our data are also generally in agreement with those from previous studies that examined cell-type expression of a limited number of genes (Dataset S3).

### Asymmetric expression profiles in gonidial *vs.* somatic cells

**Figure 3:**
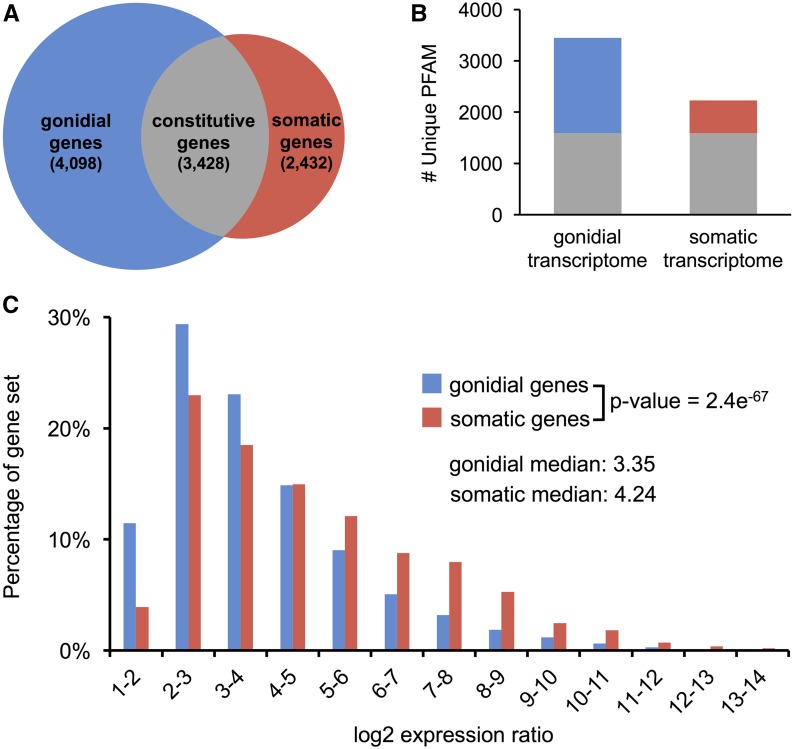
Characterization of *V. carteri* germ and somatic transcriptomes. (A) Venn diagram showing the numbers of gonidial genes (G/S > 2) in blue, constitutively expressed genes in gray, and somatic genes (S/G > 2) in red. (B) Numbers of different PFAM domain identifiers associated with the gonidial and somatic transcriptomes are shown as stacked bar plots, and color coded as in (A). (C) Histograms of expression ratios (G/S or S/G) of gonidial *vs.* somatic genes. p-value of Wilcoxon rank sum test for the two distributions is indicated. The median log_2_ expression ratio for each gene set is also indicated. G, gonidial expression; S, somatic expression.

We found that over 6500 genes in *V. carteri* (∼50% of the expressed transcriptome) had significantly different expression levels between cell-types (Figure 3A, and Figure S2 and Dataset S1). Comparative metrics of cell-type expression patterns further revealed unanticipated asymmetry between the two cell-type transcriptomes. First, there were almost 1.7 times more gonidial genes (4098) than somatic genes (2432) (Figure 3A), with significantly more PFAM domains encoded by the gonidial transcriptome, both of which indicate greater gonidial *vs.* somatic transcriptome diversity (Figure 3B). On the other hand, somatic transcripts had a significantly higher degree of cell-type specificity (*i.e.*, magnitude of expression bias) than gonidial transcripts (Figure 3C), indicating a greater degree of somatic cell-type specialization. Taken together, these findings demonstrate that the somatic transcriptome is more specialized than the gonidial transcriptome: somatic cells express fewer genes than gonidial cells, and the genes that are expressed in somatic cells are expressed in a more cell-type-specific manner. This somatic cell specialization pattern was retained even when genes related to flagella (Dataset S4) were removed from the analysis (Figure S3 in File S1), suggesting that specialization of the somatic transcriptome extends beyond flagella gene expression, which was examined in more detail below.

### Functional classification of gonidial and somatic genes

We used MapMan annotations (Thimm *et al.* 2004), which were derived from *C. reinhardtii* genes and assigned to *V. carteri* genes based on orthology, to search for functional enrichment in the proteins encoded by the *V. carteri* gonidial and somatic transcriptomes (*Materials and Methods*). From 8763 functional assignments in *V. carteri* we identified 12 categories that were significantly enriched among gonidial genes, most of which were associated with biosynthetic processes including DNA synthesis, RNA processing, amino acid synthesis, protein assembly/cofactor ligation, and tetrapyrrole synthesis (Table S3 in File S1). In contrast, only three categories were enriched in somatic genes: motility (related to flagella genes), cell organization (also found among flagella/cytoskeletal related genes), and post-translational modification (mostly protein kinases and phosphatases). GO terms enriched in each gene set were consistent with MapMan enrichments, with signal transduction-associated functions also found to be enriched in the somatic gene set (Table S4 in File S1). Together, these classification data validate the known properties of the two cell-types, with gonidia enriched for expression of biosynthetic functions related to cell growth and somatic cells enriched for expression of motility functions.

### Evolutionary origins of germ and somatic genes

**Figure 4:**
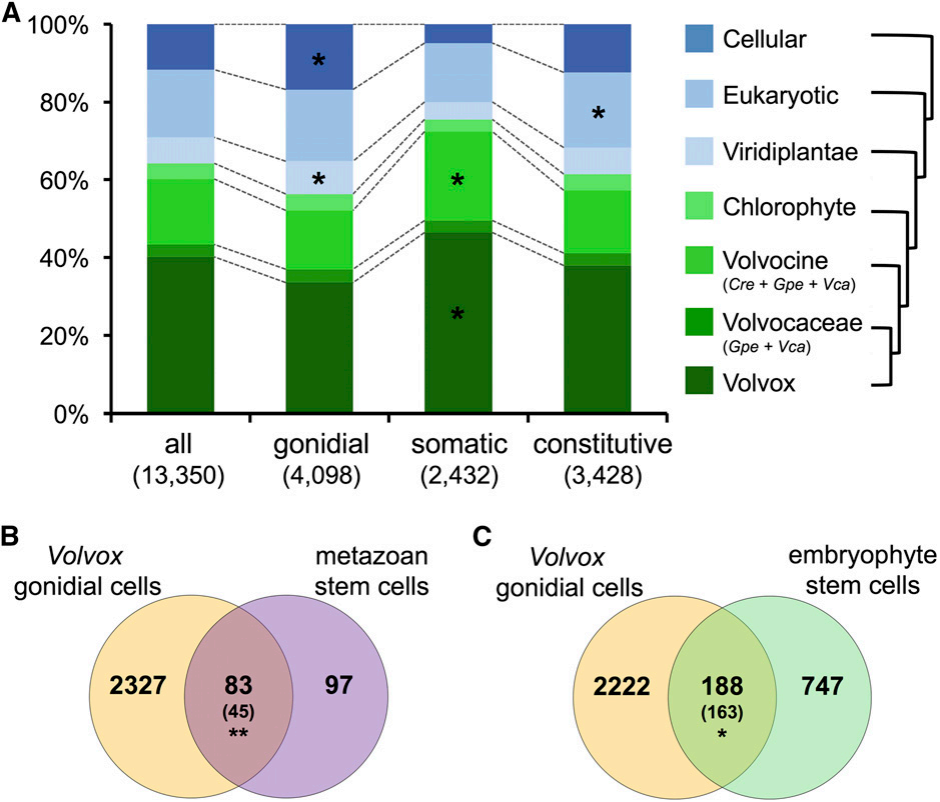
Evolutionary genomics analyses of gonidial and somatic genes. (A) Phylostratigraphic profiling. Bar graphs show the fraction of genes from each *V. carteri* cell-type expression classification group in each phylostratum. Color key on right shows nested phylostrata progressing from the most recent phylostratum of *V. carteri*-specific genes on the bottom to the most ancient phylostratum of genes found across all cellular organisms at the top. Asterisk indicates significant enrichment compared to all expressed genes (* FDR < 0.01). (B and C) Venn diagrams showing overlap between OrthoMCL IDs associated with *V. carteri* gonidial cells and animal (metazoan) stem cells (B) or land plant (embryophyte) stem cells (C). Expected values are in parentheses. Asterisks indicates significant enrichment (** FDR < 0.01 and * FDR < 0.05). Cre, *C. reinhardtii*; FDR, false discovery rate; Gpe, *Gonium pectorale*; IDs, identifiers; Vca, *V. carteri*.

We next investigated the evolutionary origins of cell-type specialization in *V. carteri* using phylostratigraphy (Domazet-Loso *et al.* 2007; Domazet-Loso and Tautz 2010; Lopez *et al.* 2015; Hanschen *et al.* 2016). Briefly, well-annotated proteomes were collected from species with increasing divergence times from *V. carteri* and were grouped into a nested set of taxonomic categories or phylostrata: *V. carteri* (*V. carteri* only), Volvocaceae (multicellular volvocine algae: *G. pectorale* + *V. carteri*), all volvocine algae (*C. reinhardtii* + *G. pectorale* + *V. carteri*), chlorophyte green algae, viridiplantae (chlorophyte algae + streptophytes), all eukaryotes, and all cellular organisms (eukaryotes + prokaryotes + archaea) (Table S5 in File S1). The phylogenetic origin of each *V. carteri* protein-coding gene was then determined by identifying the most distantly related phylostratum that contained a homolog based on BLASTP searches that were customized for long-distance comparisons (*Materials and Methods*). Compared to the entire transcriptome, gonidial genes were enriched for those with ancient origins (genes conserved across all cellular organisms) and those originating within green eukaryotes (Viridiplantae). In contrast, somatic genes showed a reciprocal phylostratigraphic pattern, with a strong enrichment for lineage-specific genes that were found only in *V. carteri* or only in the volvocine algae (Figure 4A). Constitutively- expressed genes had a phylostratigraphic distribution similar to the background transcriptome, though they showed a modest enrichment for genes conserved across eukaryotes. Interestingly, no enrichments were observed for genes found only among the multicellular Volvocine algae (*G. pectorale* + *V. carteri*). Together, our comparative phylostratigraphy data show that somatic cells preferentially express a more derived set of lineage-specific genes, many of which arose in the most recent unicellular ancestor of *V. carteri*, while gonidial genes encode more deeply ancestral and conserved functions.

The older and more conserved origins of gonidial genes prompted us to compare the genetic program of *V. carteri* gonidia, a pluripotent cell-type, with the genetic programs of pluripotent cells from metazoans and land plants (embryophytes), both of which have independently evolved multicellularity. A previous study identified a core set of metazoan pluripotent stem cell genes based on shared OrthoMCL classifications among early-diverging animal taxa (Chen *et al.* 2006; Alié *et al.* 2015). We extended this analysis to include *V. carteri* cell-type genes and embryophyte apical stem cell genes that were previously found to be shared between maize shoot apical meristems and moss (*P. patens*) gametophore apical cells (Frank and Scanlon 2015) (*Materials and Methods*) (Figure 4, B and C). In this analysis we found a significant nearly twofold enrichment of OrthoMCL IDs shared between *V. carteri* gonidia and metazoan stem cells (83 observed *vs.* 45 expected) (Figure 4B), suggesting that these two lineages converged on similar networks of pluripotency-associated genes. There was also a modest but significant enrichment of OrthoMCL IDs shared between *V. carteri* gonidia and embryophyte stem cells (188 observed *vs.* 163 expected), whose total overlap was larger than with metazoan stem cells (188 *vs.* 83 IDs) but whose relative enrichment was less pronounced (Figure 4C). Interestingly, the greatest enrichment between *V. carteri* gonidia and embryophyte stem cells was in the subset of OrthoMCL IDs that were also shared with metazoan stem cells (Figure S4A in File S1). In contrast, most of the *V. carteri*–metazoan overlap for stem cell IDs was not shared with embryophytes. Additional two-way comparisons between metazoan or embryophyte stem cells with different *V. carteri* expression groups (somatic and constitutive + low-confidence) yielded complementary results, where we found strong deenrichment for overlap of *V. carteri* somatic cell OrthoMCL IDs and metazoan or embryophyte stem cell OrthoMCL IDs (Figure S4, B and C in File S1). *V. carteri* genes with little or no expression bias (constitutive + low-confidence genes) had modest deviations from expected OrthoMCL ID overlaps, showing deenrichment with metazoan stem cells and enrichment in embryophyte stem cells (Figure S4, D and E in File S1). Taken together, our data suggest commonality and convergence between stem cell genetic networks across the three multicellular lineages, with *V. carteri* gonidia and metazoan stem cells having retained a more significant proportion of overlap despite their greater divergence time, and with a larger magnitude of overlap between *V. carteri* gonidia and embryophyte apical stem cells. We also note that the metazoan stem cell data set of 180 OrthoMCL IDs (Alié *et al.* 2015) was more stringently derived than the embryophyte stem cell data set (935 OrthoMCL IDs) (Frank and Scanlon 2015), making it difficult to draw firm conclusions about the relative similarities among the three stem cell comparison groups. Nonetheless, our study shows that a comparative genomics approach to stem cell evolution may help refine our understanding of the origins and maintenance of pluripotency in different multicellular lineages.

In the following sections, we describe cellular and metabolic processes or pathways that are differentially expressed between gonidial and somatic cells, and which highlight the functional specializations of each cell-type. We then test a more general model of cooption based on conversion of temporally segregated gene expression patterns in a unicellular ancestor to cell-type segregated expression patterns in *V. carteri*.

### Distinct gene expression patterns of flagellar, basal body, and transition zone (TZ) genes

**Figure 5:**
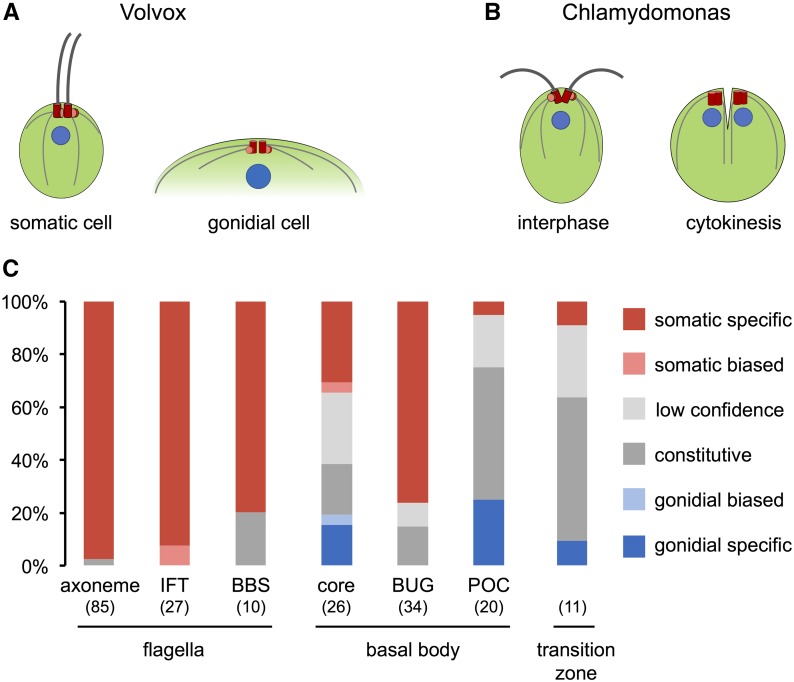
Expression patterns of flagella, basal body, and transition zone genes. (A) Cartoon diagrams of the cytoskeletal apparatus from a *V. carteri* somatic cell (left) and the apical portion of a *V. carteri* gonidial cell (right). Flagella and microtubule rootlets are shaded dark and light gray, respectively, nuclei are shaded blue, mature basal bodies are shaded red, and probasal bodies shaded pink/red. (B) Cartoon diagrams of interphase (left) and dividing (right) *C. reinhardtii* cell shaded as described in (A). Interphase basal bodies in *C. reinhardtii* are arranged with 180° symmetry, causing the flagella to beat in opposite directions, while basal bodies in a *V. carteri* somatic cell are each rotated 90° relative to those in *C. reinhardtii*, causing the flagella to beat in parallel. During cytokinesis, *C. reinhardtii* cells resorb flagella and basal bodies function as centrioles to coordinate positioning of the cleavage furrow between daughter nuclei. (C) Stacked bar graph shows the fraction of flagella-related genes [axonemal genes, intraflagellar transport (IFT) genes, and Bardet–Biedel Syndrome (BBS) genes], basal body genes (core genes with validated function/localization, BUG genes, and POC genes), and transition zone genes (see Dataset S4) in each cell-type expression classification shown in the color key on the right. Number of genes for each group is in parentheses.

Besides their vastly different sizes, a distinctive derived feature of *V. carteri* cell-types is the absence and presence of flagella on gonidia and somatic cells, respectively (Figure 5A). In *C. reinhardtii*, flagella genes are coexpressed as a tightly coordinated regulon that is activated after flagella loss or during the cell cycle after flagella resorption and cell division (Stolc *et al.* 2005; Chamberlain *et al.* 2008; Albee *et al.* 2013; Zones *et al.* 2015). It seemed possible that the entire flagella regulon of *C. reinhardtii* might be conserved in *V. carteri* and was coopted to be active only in somatic cells. Alternatively, suppressed expression of one or a few key flagella biogenesis genes in gonidia could also completely block flagella formation (McVittie 1972; Huang *et al.* 1977; Huskey *et al.* 1979; Huskey 1979; Adams *et al.* 1982; Tam and Lefebvre 1993; Rosenbaum and Witman 2002). A third possibility is that flagella biogenesis is controlled post-transcriptionally in *V. carteri*, in which case no significant differences in flagella-related mRNA abundance would be observed between cell-types. As shown in Figure 5C, nearly all flagella genes in *V. carteri* were somatic-specific, a result that is consistent with the flagella regulon cooption model (Figure 5C and Dataset S4).

Flagella biogenesis also requires basal bodies that template axoneme assembly, position the flagella at the cell’s anterior pole, and organize the internal microtubule cytoskeleton (Dutcher 2003; Marshall 2008). In ciliated or flagellated cells, including *C. reinhardtii*, basal bodies have dual functions that are mutually exclusive: during interphase they template the assembly of cilia/flagella, while during cell division they replicate and help spatially coordinate mitosis and cytokinesis (Figure 5B) (Coss 1974; Ehler *et al.* 1995; Marshall and Rosenbaum 2000; Simons and Walz 2006; Dawe *et al.* 2007; Parker *et al.* 2010). Since gonidia are flagella-less, yet undergo cell division, and somatic cells are motile, yet do not divide, expression of different basal body gene subsets corresponding to cell division or flagella biogenesis functions might also be segregated between the cell-types. Most of the core basal body genes (those with validated basal body function) did not show a clear pattern of preferential cell-type expression, with many being constitutive or low confidence (Figure 5C). We also searched for cell-type expression biases among *V. carteri* orthologs of less well-characterized candidate basal body genes identified through two proteomics surveys in *C. reinhardtii* (Keller *et al.* 2005, 2009). Candidate basal body proteins from these studies were split into two sets based on validation methods: BUG genes were upregulated during flagellar assembly and may be related to flagella function, while POC genes were conserved in species with centrioles and are expected to be related to centriolar and cell division functions (Keller *et al.* 2005, 2009). Consistent with these validation methods, most BUG genes in *V. carteri* were preferentially expressed in somatic cells where flagella functions dominate, while most POC genes in *V. carteri* were either constitutively expressed or preferentially expressed in gonidia where core replication and mitotic functions of basal bodies are required (Figure 5C). These data suggest that cell-type expression patterns in *V. carteri* could be useful as a functional filter for distinguishing flagella/motility-related functions and cell division/replication-related functions of basal body proteins.

Lastly, we examined the cell-type expression patterns of genes encoding proteins of the TZ, a distinct region near the base of each flagellum that is connected to the plasma membrane by transition fibers and which is thought to serve as a protein diffusion barrier between the flagellum and cytosol (Craige *et al.* 2010; Awata *et al.* 2014; Dutcher and O’Toole 2016). In *C. reinhardtii*, the TZ protein-encoding genes were expressed with high periodicity just prior to or during cell division (Zones *et al.* 2015). Interestingly, *V. carteri* orthologs of most *C. reinhardtii* TZ genes were expressed at similar levels in somatic and gonidial cells, suggesting a requirement for TZ protein function in both cell-types (Figure 5C). The expression of TZ genes in aflagellate gonidia may reflect a cryptic requirement for a TZ or related structure in this cell-type, a need to stockpile TZ proteins prior to their deployment in the next generation of somatic cells, or additional nonflagella-specific roles for TZ proteins (*e.g.*, vesicle trafficking), as was predicted in *C. reinhardtii* (Diener *et al.* 2015).

### Differential photosynthetic gene expression in gonidial and somatic cells

**Figure 6:**
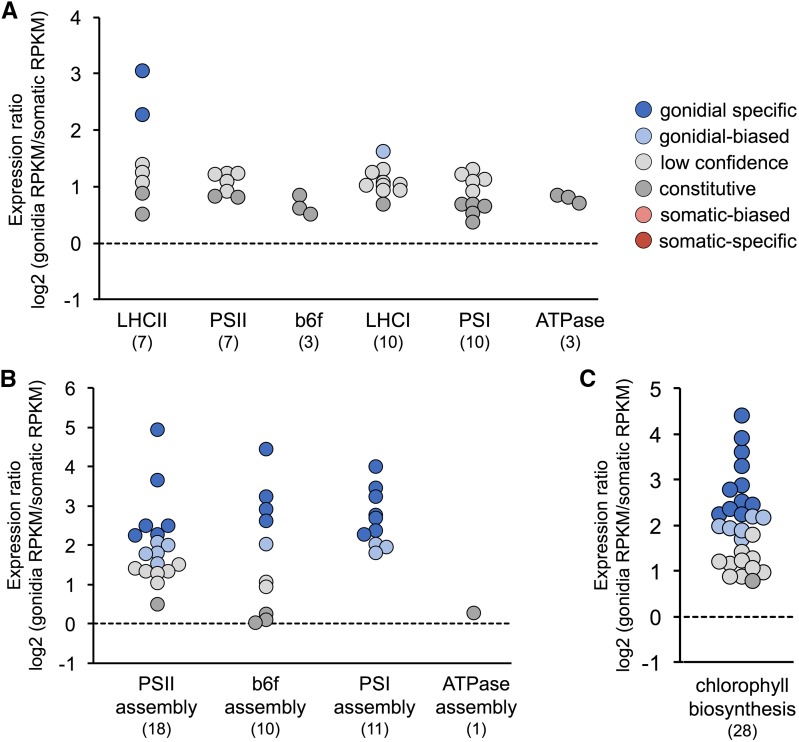
Cell-type expression patterns of photosynthesis-related genes. Each dot represents a single gene (see Dataset S5) whose expression category is color-coded according to the key at the top right. *y*-axis is log_2_-transformed gonidial/somatic expression ratio. Dashed line demarcates a 1:1 gonidial/somatic expression ratio. Numbers of genes for each subgroup are in parentheses. (A) Subunits of indicated complexes carrying out photosynthetic light reactions. (B) Assembly factors for indicated photosynthetic complexes. (C) Chlorophyll biosynthesis enzymes. RPKM, reads per kilobase per million mapped reads.

Previous studies of cell-type gene expression in *V. carteri* led to a hypothesis that photosynthetic gene expression is suppressed in somatic cells, which in turn blocks or slows cell growth and thereby ensures terminal differentiation (Tam and Kirk 1991; Choi *et al.* 1996; Meissner *et al.* 1999). We revisited this hypothesis by examining the cell-type expression patterns of genes in *V. carteri* that are predicted to encode photosynthesis-related proteins. In our transcriptome data, nearly all core photosynthetic complex genes corresponding to subunits of LHCII, photosystem II (PSII), cytochrome b6f complex (b6f), LHCI, photosystem I (PSI), and ATP synthase (ATPase) were expressed at high levels in both cell-types, and were classified as constitutive or low confidence, demonstrating that they did not show strong cell-type expression biases (Figure 6A and Dataset S5). Nonetheless, collectively they exhibited a clear pattern of 1.5–2.5-fold higher expression in gonidia than in somatic cells, suggesting that photosynthesis genes are expressed with a modest gonidial preference, but are not strongly cell-type regulated as suggested by earlier studies. Exceptions to this general finding included one major LHCII gene family (subclade I containing 19 paralogs) and a minor LHCII gene (*LHCB7*) having gonidial-specific expression patterns, and a variant LHCI paralog, *LHCA3.2*, which is found in *V. carteri* but not *C. reinhardtii*, having gonidial-biased expression (Figure S5 and Dataset S5). These exceptions might indicate differential fine-tuning of photosynthetic antenna function between *V. carteri* cell-types. In contrast to the genes encoding core subunits of photosynthetic complexes, many genes encoding photosynthetic complex assembly factors and chlorophyll biosynthesis enzymes were gonidial-biased or gonidial-specific in expression (Figure 6, B and C), suggesting a greater requirement for photosynthetic complex assembly and/or repair in gonidial cells.

**Figure 7:**
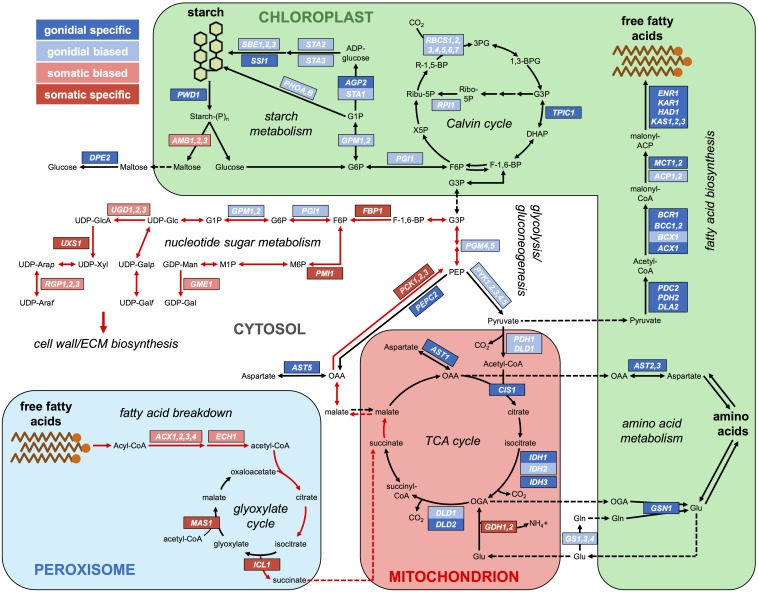
Cell-type expression patterns of carbon metabolism-related genes in *V. carteri*. Major pathways of central carbon metabolism are depicted as flow diagrams. Large shapes with colored shading and colored text labels are used to indicate subcellular compartments where indicated metabolic steps occur (chloroplast, mitochondrion, peroxisome, and cytosol). Major carbon sinks of starch and free fatty acids are also cartooned. Metabolites are in black lettering, chemical reactions are represented by solid arrows, and metabolite shuttling between subcellular compartments is represented by dashed arrows. Red arrows indicate a putative pathway for sugar biosynthesis in somatic cells. Genes encoding enzymes for indicated steps are in white lettering and surrounded by boxes that are color-coded according to their germ–soma expression patterns (color key in the upper left). Only genes with differential germ–soma gene expression are shown. Genes encoding subunits of a protein complex are grouped together in larger boxes. Paralogs inferred to have similar functions are grouped together in single boxes and separated by commas. Cell-type expression patterns of grouped paralogs were determined by computing the ratios of summed expression levels of paralogs in each cell-type (see *Materials and Methods*; Datasets S6 and S7). ECM, extracellular matrix; TCA, tricarboxylic acid.

Most genes encoding enzymes of the photosynthetic dark reactions [also known as the Calvin–Benson–Bassham (CBB) cycle] did not exhibit strong cell-type expression patterns, though the small subunits of the Rubisco enzyme complex (*RBCS1–RBCS7*), collectively showed a modest twofold gonidial bias in expression (Figure 7 and Dataset S6). Two other genes encoding CBB enzymes, *RPI1* and *TPIC1*, were also found to be preferentially expressed in gonidial cells. In summary, our data for photosynthetic gene expression do not support the hypothesis that photosynthesis is strongly suppressed in somatic cells, but do suggest a greater requirement for photosynthetic complex assembly and/or repair in gonidia.

### Carbon metabolism gene expression in germ and somatic cells

To gain further insight into the metabolic bases of germ–soma differentiation, we investigated the cell-type expression patterns of genes encoding enzymes involved in carbon metabolism (*Materials and Methods*; Dataset S6). Here, we found a clear dichotomy between carbon storage anabolism and catabolism in the two cell-types (Figure 7). Two major sinks for fixed carbon in green algae are fatty acids and starch. Nearly all genes encoding enzymes for fatty acid biosynthesis, starting with those for the plastidic pyruvate dehydrogenase complex that generates acetyl-CoA, were gonidial-specific. Conversely, genes encoding the fatty acid breakdown enzymes acyl-CoA oxidase (*ACX1-ACX4*) and enoyl-CoA hydratase (*ECH1*) were either somatic-specific or somatic-biased. A similar anabolic/catabolic dichotomy was observed for starch metabolism with preferential expression of genes for starch biosynthetic enzymes in gonidial cells—most notably *STA1* that encodes ADP-glucose pyrophosphorylase, which catalyzes the rate-limiting step for starch accumulation (Ball *et al.* 1991; Zabawinski *et al.* 2001). Conversely, genes encoding amylase enzymes (*AMB1-AMB3*) that catalyze the hydrolysis of starch were preferentially expressed in somatic cells.

Glycolysis and gluconeogenesis share most enzymes, but differ in two key steps: interconversion of fructose-6-phosphate and fructose-1,6-bisphosphate, which is catalyzed by either phosphofructokinase (PFK, glycolysis) or fructose-1,6-bisphosphatase (FBP, gluconeogenesis); and synthesis/breakdown of phosphoenolpyruvate, which is catalyzed by pyruvate kinase (PYK, glycolytic breakdown into pyruvate) or phosphoenolpyruvate carboxykinase (PCK, gluconeogenic synthesis from oxaloacetate). *PYK* and *PCK* genes showed opposing cell-type expression patterns, with *PYK* genes being gonidial-biased and *PCK* genes being somatic-specific. *PFK* did not show cell-type preference, but *FBP1* was somatic-specific (Figure 7). Taken together, these profiles suggest opposing directions of net carbon flow through glycolysis in gonidia and through gluconeogenesis in somatic cells.

Consistent with the glycolysis/gluconeogenesis gene expression dichotomy, we found that expression of TCA cycle and glyoxylate cycle genes was also cell-type-biased. Genes encoding enzymes or subunits of complexes that catalyze the first four out five reactions specific to the TCA cycle—pyruvate dehydrogenase (*PDH1* and *DLD1*), citrate synthase (*CIS1*), isocitrate dehydrogenase (*IDH1–IDH3*), and oxoglutarate dehydrogenase (*DLD1–DLD2*)—were expressed preferentially in gonidia (Figure 7 and Dataset S6). TCA cycle intermediates are also used for amino acid biosynthesis, and genes encoding enzymes that catalyze two major amino acid biosynthetic entry points, glutamate synthase (*GSN1*) and aspartate aminotransferase (*AST1–AST3* and *AST5*), were also gonidial-specific. Conversely, glutamate dehydrogenase paralogs (*GDH1* and *GDH2*), which function in amino acid catabolism, had somatic-specific expression. The glyoxylate cycle makes use of some TCA cycle enzymes but bypasses the decarboxylation steps, and is often coupled to fatty acid catabolism and gluconeogenesis to produce sugars (Eastmond and Graham 2001; Graham 2008). Genes encoding the two dedicated enzymes of the glyoxylate cycle, isocitrate lyase (*ICL1*) and malate synthase (*MAS1*), were expressed specifically in somatic cells, suggesting preferential use of the glyoxylate cycle over the TCA cycle. Our finding that genes encoding enzymes involved in fatty acid breakdown, the glyoxylate cycle, and gluconeogenesis were all preferentially expressed in somatic cells suggests that these pathways function coordinately to promote sugar biosynthesis in somatic cells (Figure 7, red arrows; see *Discussion*).

Together, our analyses of central carbon metabolism gene expression are consistent with a well-orchestrated dichotomy in metabolism between *V. carteri* cell-types, with gonidial cell metabolism geared towards cell growth and anabolism, and somatic cell metabolism geared towards starch breakdown and conversion of fatty acids into sugar through the glyoxylate cycle and gluconeogenesis.

### The sink for somatic cell sugar biosynthesis may be the extracellular matrix

Because somatic cells showed relatively lower expression of starch biosynthetic enzymes and higher expression of starch hydrolysis enzymes compared with gonidial cells (Figure 7 and Dataset S6), it seemed unlikely that sugars derived from gluconeogenesis in somatic cells would be used for starch biosynthesis. An alternative sink for sugars in somatic cells is the extracellular matrix (ECM), which comprises the vast majority of the spheroid volume (>99%) and which is composed primarily of glycoproteins (Kirk *et al.* 1986; Sumper and Hallmann 1998; Hallmann 2003). If the ECM is the sugar sink of somatic cells, then we might expect to see genes involved in protein glycosylation and genes encoding ECM glycoproteins to be expressed preferentially in somatic cells compared to gonidial cells. We first examined genes encoding enzymes involved in the biosynthesis of nucleotide sugars (UDP-xylose, UDP-arabinose, UDP-galactose, UDP-glucose, and GDP-mannose) that serve as sugar donors for cell wall/ECM glycosylation in volvocine algae (Sumper and Hallmann 1998; Hallmann 2003; Seifert 2004; Harris 2009). Genes encoding five nucleotide sugar biosynthesis enzymes—phosphomannose isomerase (*PMI1*), GDP-D-mannose 3′, 5′-epimerase (*GME1*), UDP-glucose dehydrogenase (*UGD1-UGD3)*, UDP-xylose synthase (*UXS1*), and UDP-arabinopyranose mutase (*RGP1-RGP3*)—were expressed preferentially in somatic cells (Figure 7 and Dataset S7). The only genes involved in nucleotide sugar biosynthesis that were expressed preferentially in gonidia—phosphoglucose isomerase (*PGI1*) and phosphoglucomutase (*GPM1* and *GPM2*)—also play a central role in starch biosynthesis, which is likely to be preferentially expressed in gonidia, as described above (Figure 7 and Dataset S6).

**Figure 8:**
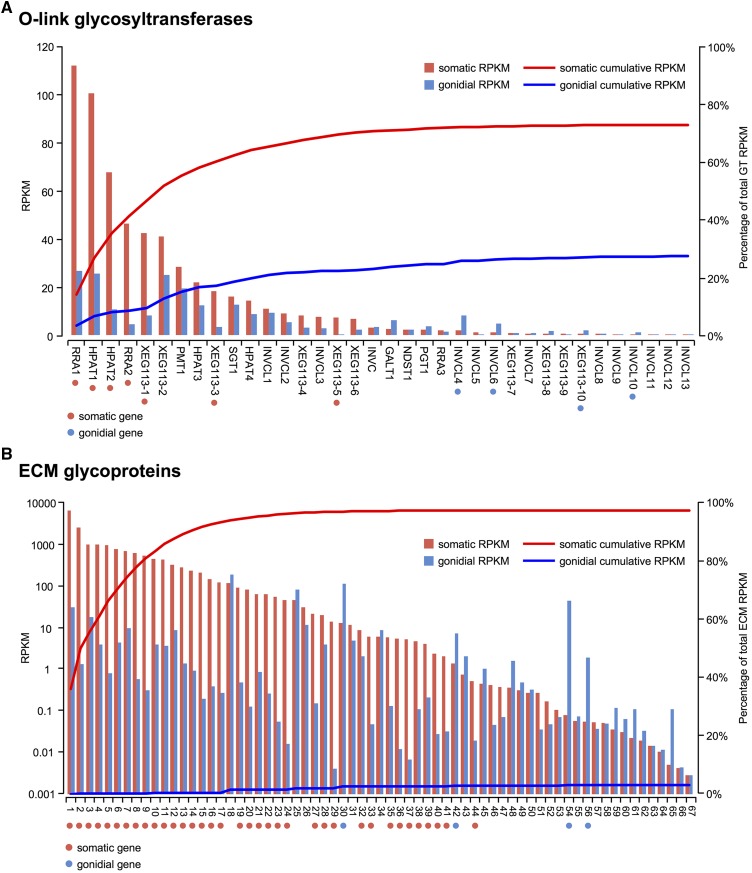
*V.* *carteri* ECM-related gene expression. Combined bar and cumulative expression plots of somatic (red) and gonidial (blue) expression for genes encoding predicted glycosyltransferases (GTs) involved in O-linked glycosylation (A) (Dataset S8) or ECM-targeted glycoproteins (B) (Dataset S9). Bars indicate expression levels of genes in RPKM (*y*-axis, left side) in each cell-type and lines indicate cumulative expression in each cell-type, summed across all genes (left to right) as percentages of total expression (*y*-axis, right side). Note that the *y*-axis for RPKMs in (B) is log-scaled. Red and blue dots mark somatic and gonidial genes, respectively. ECM, extracellular matrix; RPKM, reads per kilobase per million mapped reads.

We also assessed expression patterns of genes encoding glycosyltransferases that are involved in O-linked glycosylation and are predicted to glycosylate the hydroxyproline-rich glycoproteins (HRGPs) that comprise most of the *V. carteri* ECM (*Materials and Methods*) (Sommer-Knudsen *et al.* 1998; Sumper and Hallmann 1998; Hallmann 2003; Showalter and Basu 2016). Out of the 36 predicted glycosyltransferase-encoding genes in *V. carteri*, seven were somatic and four were gonidial. However, >73% of total glycosyltransferase gene expression summed over all 36 genes was found in somatic cells, suggesting a higher demand for this activity in soma (Figure 8A and Dataset S8).

Finally, we assessed cell-type expression patterns of predicted ECM glycoprotein genes and found that 36/67 genes were somatic, while only 4/67 were gonidial. Moreover, >95% of total ECM gene expression (summed expression across all ECM genes) was in somatic cells (Figure 8B and Dataset S9).

To test more generally whether somatic cells might be specialized for secretion, we predicted the subcellular localization of all *V. carteri* proteins (*Materials and Methods*) and then grouped them with respect to cell-type expression pattern. In doing so, we found a significant enrichment of predicted secreted or endomembrane-targeted proteins among somatic genes, while gonidia were enriched for expression of predicted chloroplast- and mitochondrial-targeted proteins (Figure S6 in File S1). Together, our findings suggest that somatic cells are specialized for the production and secretion of ECM glycoproteins, and that a major sink for somatic cell sugars is the ECM.

### Testing the temporal–spatial cooption hypothesis for the origin of cell-type gene expression in V. carteri

**Figure 9:**
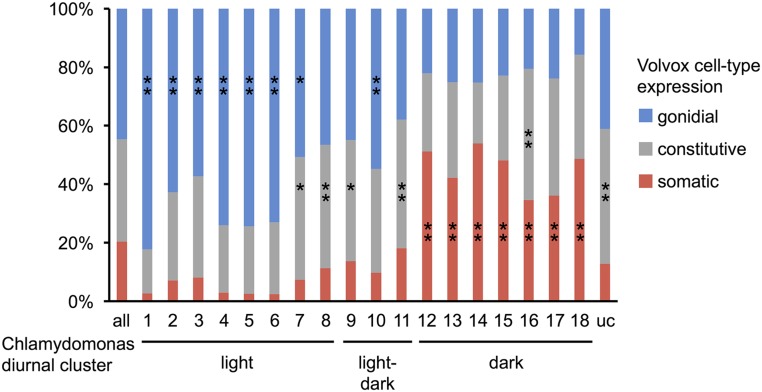
Cell-type expression patterns of *V. carteri* orthologs of diurnally expressed *C. reinhardtii* genes. Stacked bar plots show fractions of *V. carteri* genes from each cell-type expression classification whose corresponding orthologs in *C. reinhardtii* belong to the indicated diurnal cluster [all genes, c1–c18, or unclustered (uc) (Zones *et al.* 2015)]. Clusters are arranged in temporal order with peak expression within the light-, light–dark transition, or dark- phase of the diurnal cycle indicated below groups of clusters. Color key is shown to the right. Significant enrichment of *V. carteri* cell-type expression within each *C. reinhardtii* diurnal cluster is indicated as follows: ** FDR < 0.01 and * FDR < 0.05.

As detailed above, *V. carteri* genes related to flagellar motility, photosynthesis, central carbon metabolism, and ECM secretion exhibited coherent expression patterns, with many showing a clear germ–soma dichotomy. Interestingly, *C. reinhardtii* orthologs for most of these genes showed strong and coordinate periodic expression during a diurnally synchronized cell cycle (Zones *et al.* 2015). This correlation between cell-type expression patterns in *V. carteri* and diurnal expression patterns in *C. reinhardtii* suggests that cell-type gene expression programs in *V. carteri* might have originated through cooption of preexisting temporal expression programs in a *C. reinhardtii*-like unicellular ancestor. To more directly test for temporal–spatial cooption, we investigated the relationship between diurnal gene expression in *C. reinhardtii* and cell-type gene expression in *V. carteri*. We identified *V. carteri* orthologs of *C. reinhardtii* genes from previously characterized diurnal gene expression clusters (Zones *et al.* 2015) and assessed their cell-type expression patterns. If temporal clustering in *C. reinhardtii* were unrelated to cell-type expression in *V. carteri*, then we would expect to see a similar representation of *V. carteri* expression patterns within each *C. reinhardtii* temporal cluster. On the contrary, we found a strong relationship between diurnal gene expression in *C. reinhardtii* and cell-type gene expression in *V. carteri*: *C. reinhardtii* genes belonging to light-phase clusters (c1–c8) were enriched for genes whose *V. carteri* orthologs were gonidial, while *C. reinhardtii* genes belonging to dark-phase clusters (c12–c18) were enriched for genes whose *V. carteri* orthologs were somatic (Figure 9). We performed a reciprocal version of this enrichment test by first grouping *C. reinhardtii* diurnal genes into four major superclusters (light-phase, light/dark transition, dark-phase, and unclustered) (*Materials and Methods*), and then determined the composition of each supercluster based on orthology to *V. carteri* cell-type genes. Again, we found significantly skewed distributions where *C. reinhardtii* orthologs of *V. carteri* gonidial genes were enriched for light-phase supercluster assignments, while *C. reinhardtii* orthologs of *V. carteri* somatic genes were enriched for dark-phase supercluster assignments (Figure S7A in File S1). These results were essentially unchanged when we removed flagella-associated genes from the analysis, indicating that the diurnal and cell-type expression patterns of the ∼120 flagella-related genes in the two species (Dataset S4) were not solely responsible for the results we observed supporting cooption (Dataset S4) (Figure S7B in File S1). Together, our results are consistent with large-scale cooption, where regulons that exhibited diurnal regulatory patterns in a unicellular ancestor came under cell-type control in *V. carteri*.

## Discussion

Although germ–soma differentiation has been studied extensively in animals and plants, very little is known about the early evolution of germ–soma specialization, especially the nature of genetic programming required to initially achieve this fundamental cell-type dichotomy. As discussed below, our transcriptome data from *V. carteri* has yielded insights about this key innovation that may apply more broadly to other multicellular lineages outside of volvocine algae.

### An asymmetric germ–soma dichotomy may be a convergent trait

Our data revealed not only extensive differential expression between *V. carteri* cell-types (50% of expressed genes were cell-type-regulated), but also an unanticipated asymmetry in expression patterns. *V. carteri* germ cells, though specialized for reproduction, are more generalist in their overall genetic programming as they express a significantly larger transcriptome than somatic cells, but with less overall expression bias (*i.e.*, degree of cell-type specificity) (Figure 3). We also found that gonidia express a more conserved and ancient set of genes than do somatic cells (Figure 4A). This generalist and ancestral nature of the gonidial transcriptome may be a convergent trait in the transcriptomes of pluripotent cell-types. Similar to *V. carteri*, pluripotent embryonic stem cells in mammals and pluripotent adult stem cells in early diverging metazoan taxa express more genes than their differentiated daughter cells (Brandenberger *et al.* 2004; Miura *et al.* 2004; Denis *et al.* 2011; Hemmrich *et al.* 2012; Gan *et al.* 2014; Zhao *et al.* 2014; Alié *et al.* 2015), and preferentially express genes with phylogenetically older origins (Hemmrich *et al.* 2012; Alié *et al.* 2015). Though transcriptomic analyses have been conducted on the pluripotent stem cell populations of land plants (Nawy *et al.* 2005; Yadav *et al.* 2009, 2014; Frank and Scanlon 2015), comparable pairwise analyses of stem cells and specific differentiated daughter lineages have not been reported for land plants. For example, the land plant stem cell gene datasets used for our analyses (Figure 4, B and C and Figure S4 in File S1) were derived from overlapping genes upregulated in the stem cell populations of *Z. mays* or *P. patens* relative to whole-plant samples (Frank and Scanlon 2015). Our finding that a significant fraction of pluripotency-associated genes is conserved between *V. carteri*, animals, and land plants (Figure 4, B and C and Figure S4A in File S1) suggests potential convergent evolution of pluripotency, and may indicate a common underlying set of constraints that shaped stem cell evolution in these divergent lineages of multicellular eukaryotes.

### Lineage-specific genes and the early evolution of differentiated cell-types

All multicellular taxa trace their roots back to unicellular ancestors whose genetic toolkits were exploited and modified to solve a set of problems associated with multicellular organization, such as cell adhesion, morphogenesis, and cell-type specialization (Cock *et al.* 2010; Richter and King 2013; Umen 2014). While genes whose origins are restricted to a multicellular clade are obvious candidates for encoding multicellular innovations, genes shared by a multicellular clade and its closest unicellular relatives (but not found outside this grouping) may also have special significance for understanding the transition to multicellularity, as they represent clade-specific adaptations that arose in a common ancestor and persisted in both unicellular and multicellular descendants.

Our germ–soma transcriptome data suggests that lineage-specific genes did play a large role in the evolution of cell-type specialization in *V. carteri* where two categories of such genes were disproportionately expressed in somatic cells: (1) those that were found only in *V. carteri*, meaning that they arose in the *V. carteri* clade after the transition to simple multicellularity; and (2) those shared with *C. reinhardtii* but not elsewhere, meaning that they arose in a recent common unicellular ancestor of all volvocine algae (Figure 4A). Absent from somatic enrichment were genes found exclusively in multicellular volvocine algae (*V. carteri* + *G. pectorale*), suggesting that the program of somatic cell differentiation did not strongly rely on genes that originated during the transition to simple multicellularity (*i.e.*, typified by *G. pectorale* colonies that have undifferentiated groups of cells).

Category 1 somatic cell genes (those exclusive to *V. carteri*) evolved during the evolution of germ–soma differentiation, a trait found in two volvocine genera, *Volvox* and *Pleodorina*. While complete germ–soma differentiation is a synapomorphy in the clade containing *V. carteri* (three *V. carteri* forma plus *V. obversus*), partial germ–soma differentiation may have evolved more than once in the volvocine tree and was also likely to have been lost in some sublineages (Herron and Michod 2008). Future studies of cell-type gene expression may shed light on whether lineage-specific genes were recruited for somatic differentiation in other volvocine species where germ–soma division of labor was independently gained or lost.

Category 2 somatic cell genes (those that are pan-volvocine) presumably arose as genetic specializations in a unicellular ancestor, and were subsequently coopted or redeployed for somatic cell differentiation. Our finding that a large fraction of cell differentiation genes in *V. carteri* originated in a close unicellular ancestor parallels findings in metazoans, where much of the apparently specialized genetic toolkit associated with animal multicellularity also first evolved in unicellular ancestors that were similar to present-day choanoflagellates (Rokas 2008; Ruiz-Trillo *et al.* 2008; Richter and King 2013). Our data not only help to generalize the relationship between unicellular clade-specific innovations and multicellular innovation, but further suggest that the earliest stages of cell-type specialization may be strongly dependent on lineage-specific genes that provide a functional connection between the unicellular and multicellular lifestyles (Figure 4A). For example, *V. carteri* somatic cells resemble free-living, nonmitotic, *C. reinhardtii* cells just as choanocytes—a specialized cell-type in multicellular sponges—resemble free-living extant choanoflagellates (Kochert and Olson 1970; Coleman 2012; Mah *et al.* 2014). Fewer data are available for land plants and their closest unicellular relatives, the charophyte algae, but several studies indicate that the genetic bases for important land plant multicellular/developmental pathways, such as those for phytohormone biosynthesis and signaling, were present in early charophytes (Becker and Marin 2009; Hori *et al.* 2014; Umen 2014; Bowman *et al.* 2017).

### Cooption of temporal gene expression programs for cell-type differentiation

It has been previously proposed that the evolution of cell-type differentiation in multicellular organisms involved the cooption of different life cycle stages of a unicellular ancestor (Wolpert 1990, 1992; Nedelcu and Michod 2004; Mikhailov *et al.* 2009; Nedelcu 2012). The *C. reinhardtii* vegetative life cycle has strong temporal programming, with transitions between a motility/cell growth phase and an immotile cell division phase (Cross and Umen 2015). Koufopanou (1994) proposed a flagellar constraint hypothesis to explain the evolution of germ–soma segregation in the largest volvocine genera, where the evolution of permanently motile somatic cells allowed germ cells to escape the presumed loss-of-fitness associated with long periods of immotility during division (Koufopanou 1994). A similar trade-off between reproduction and motility has been proposed as a selective pressure for the evolution of cell-type differentiation in animals (Buss 1987; King 2004).

In our study, genes encoding flagella proteins were a clear example supporting temporal–spatial cooption, as they showed strong diurnal regulation in *C. reinhardtii* (Wood *et al.* 2012; Zones *et al.* 2015) and strong differential expression between *V. carteri* cell-types (Figure 5). More broadly, we found extensive evidence for a general pattern of temporal–spatial expression cooption based on comparisons of *C. reinhardtii* cell cycle/diurnal cycle genes (likely similar to an ancestral unicellular program) and *V. carteri* cell-type genes (Figure 9 and Figure S7 in File S1). Importantly, the *V. carteri* gonidial program was most closely associated with light-phase regulons in *C. reinhardtii*, while the *V. carteri* somatic program was most closely associated with dark-phase regulons in *C. reinhardtii*, even though our cell-type transcriptome samples were taken during the light-phase of a diurnal cycle. These differences are unlikely to reflect a complete blockage of light-based gene expression programs in somatic cells that retained relatively high levels of photosynthetic complex gene expression (Figure 6 and Dataset S5), but instead indicate a rewiring of metabolism to promote catabolic processes that typically occur in the dark phase for *C. reinhardtii* and which might also reduce or prevent net cell growth and proliferation of somatic cells. While our results support temporal–spatial cooption as a model for the origin of cell-type expression programs, this cooption could occur by a number of different mechanisms. One possibility is that temporal programs in the unicellular ancestor were converted into fixed spatial programs in *V. carteri*, such that the temporal regulatory cues of these programs were completely replaced by developmental cues. A second possibility is that the temporal programs were converted into spatial programs through phase-shifting of ancestral temporal patterns between *V. carteri* cell-types. However, phase-shifting seems like a less plausible scenario, as there are many functions that are either adaptively tied to a specific diurnal cue (*e.g.*, photosynthetic machinery expression in light) or would never be appropriate to be expressed in one cell-type *vs.* the other, regardless of relative timing (*e.g.*, cell division genes in somatic cells). A more extensive time course will be required to conclusively distinguish phase shifting *vs.* fixed expression differences between cell-types. Overall, very little is known about how diurnal and other gene expression programs are regulated in volvocine algae, though many transcription factor genes have diurnally controlled expression profiles in *C. reinhardtii* (Zones *et al.* 2015), including *RLS1* (Nedelcu 2009) whose *V. carteri* homolog, *regA*, is a master regulator of somatic cell fate (Kirk *et al.* 1999; Duncan *et al.* 2006, 2007). Our results suggest widespread cooption of temporal gene expression programs in *V. carteri* for cell-type gene expression, and further predict that some form of cell-type control has evolved for *V. carteri* orthologs of transcription factors that govern diurnal regulons in *C. reinhardtii*.

### A model for somatic cell metabolic specialization in V. carteri

Previous studies of differential gene expression in *V. carteri* led to a model for somatic cell differentiation based on suppression of photosynthetic gene expression (Tam and Kirk 1991; Choi *et al.* 1996; Meissner *et al.* 1999). Our data indicate that this model must be revised. While we did see an approximately twofold higher expression of core photosynthetic complex genes in gonidia *vs.* somatic cells (Figure 6A), photosynthetic gene transcripts were still among the most abundant in somatic cells (Dataset S5). Our results and those of a previous study (Pommerville and Kochert 1982) suggest that both *V. carteri* cell-types actively support photosynthesis, with a higher capacity in gonidial cells to support active cell growth. The elevated expression of photosynthetic complex assembly factors and chlorophyll biosynthetic enzymes in gonidia is consistent with their faster rates of growth and rates of chloroplast DNA synthesis compared to somatic cells (Coleman and Maguire 1982; Kirk 1998) (Figure 6, B and C).

**Figure 10:**
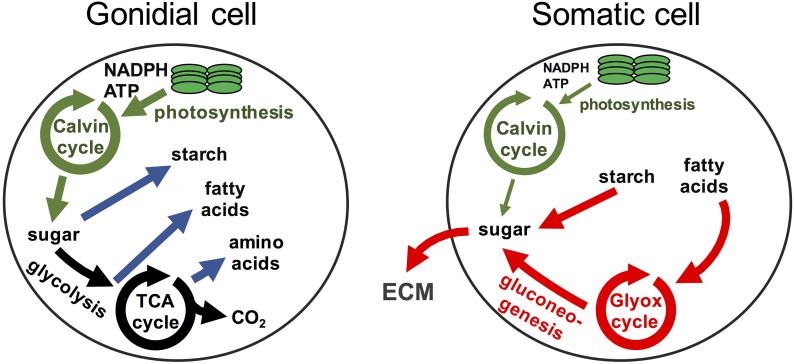
Model for gonidial and somatic cell metabolic specialization. Gonidia (left) have active photosynthesis as well as production of new photosynthetic machinery to support growth metabolism with net production of starch, lipids, and amino acids. Glycolysis and the TCA cycle are the preferred route for carbon flow in gonidial cells. Somatic cells (right) maintain active photosynthesis at somewhat reduced levels compared to gonidia, and with reduced production of new photosynthetic machinery. Lipids are broken down and metabolized through the glyoxylate cycle and gluconeogenesis to make sugars. Starch is also catabolized into sugar monomers which are used as substrates to produce excreted ECM glycoproteins. ECM, extracellular matrix; TCA, tricarboxylic acid.

While our data did not support the photosynthesis suppression hypothesis, they did suggest that gonidial and somatic cells have very different metabolic programs. Based on expression profiles, gonidial metabolism was geared toward anabolic processes such as starch, lipid, and amino acid biosynthesis, while somatic cell metabolism was programmed to couple starch and fatty acid breakdown to the glyoxylate cycle and gluconeogenesis to facilitate sugar biosynthesis (Figure 7, Figure 10, and Table S3 in File S1). The predicted somatic cell metabolic profile we inferred is similar to that of germinating oil seeds in land plants where fatty acid oxidation, the glyoxylate cycle, and gluconeogenesis are coordinately upregulated to convert stored lipids into sugars (Canvin and Beevers 1961; Runquist and Kruger 1999; Eastmond and Graham 2001; Rylott *et al.* 2001; Graham 2008). Somatic cell metabolism also resembles that of senescing leaves, where fatty acid breakdown is coupled to the glyoxylate cycle or to the TCA cycle to promote the biosynthesis and export of sugars or amino acids (Troncoso-Ponce *et al.* 2013). Autophagy is known to be induced during leaf senescence, where it is involved in breaking down chloroplasts to recycle nutrients (Schippers *et al.* 2015). An examination of *V. carteri* homologs of autophagy (ATG) genes in our study did not reveal a coherent bias toward expression in either cell-type (Figure S8 and Dataset S10), though we did see significantly higher expression of the autophagy marker gene *ATG8* in somatic cells (72 RPKM) *vs.* gonidia (9 RPKM). Because the absolute somatic expression level of *V. carteri* *ATG8* is within the range observed for rapidly proliferating *C. reinhardtii* cells (Zones *et al.* 2015), the significance of its higher expression in somatic cells *vs.* gonidia is unclear. It may be that autophagy plays a clearer or more prominent role in somatic cell biology later in the somatic life cycle, when cells are older and have exhausted nutrient stores.

Our data not only suggest a coordinated program of catabolism in somatic cells to produce sugars, but also a potential sink for those sugars as ECM glycoproteins. This idea is supported by expression data for nucleotide sugar biosynthetic genes, glycosyltransferase genes, and ECM glycoprotein genes, many of which were strongly expressed in somatic cells (Figure 7, Figure 8, and Dataset S7, S8 and S9). Thus, we propose that somatic cells—which are terminally differentiated and destined to senesce and die—slowly cannibalize themselves to generate an extensive ECM, while at the same time maintain moderately active photosynthesis to power basal metabolism, flagellar motility, and secretory activity (Figure 10). This model for germ–soma metabolic specialization in *V. carteri* provides some insight into the metabolic basis for how reproductive altruism might have evolved in the volvocine algae and possibly in other multicellular lineages. During the evolution of germ–soma differentiation, the metabolism of putative somatic cells was shifted away from cell growth-related processes and instead toward secretion that supports spheroid expansion. Secretory functions of somatic cells may also serve to support growth of gonidial cells, as previous work has shown that gonidia within intact spheroids grow faster than isolated gonidia (Koufopanou and Bell 1993). The metabolic model we propose for *V. carteri* cell-type specialization provides a framework for further investigation of how reproductive division of labor evolved within a simple multicellular organism.

## 

## Supplementary Material

Supplemental material is available online at www.g3journal.org/lookup/suppl/doi:10.1534/g3.117.300253/-/DC1.
