# Supplementary material for: Cell-Type Transcriptomes of the Multicellular Green Alga Volvox carteri Yield Insights into the Evolutionary Origins of Germ and Somatic Differentiation Programs
Source: G3 (Bethesda). 2017 Dec 5;8(2):531–50. doi: 10.1534/g3.117.300253 (PMC5919742; doi:10.1534/g3.117.300253)
Supplement: Supplementary file 2 [file 531FileS1.pdf]

Supplemental Material

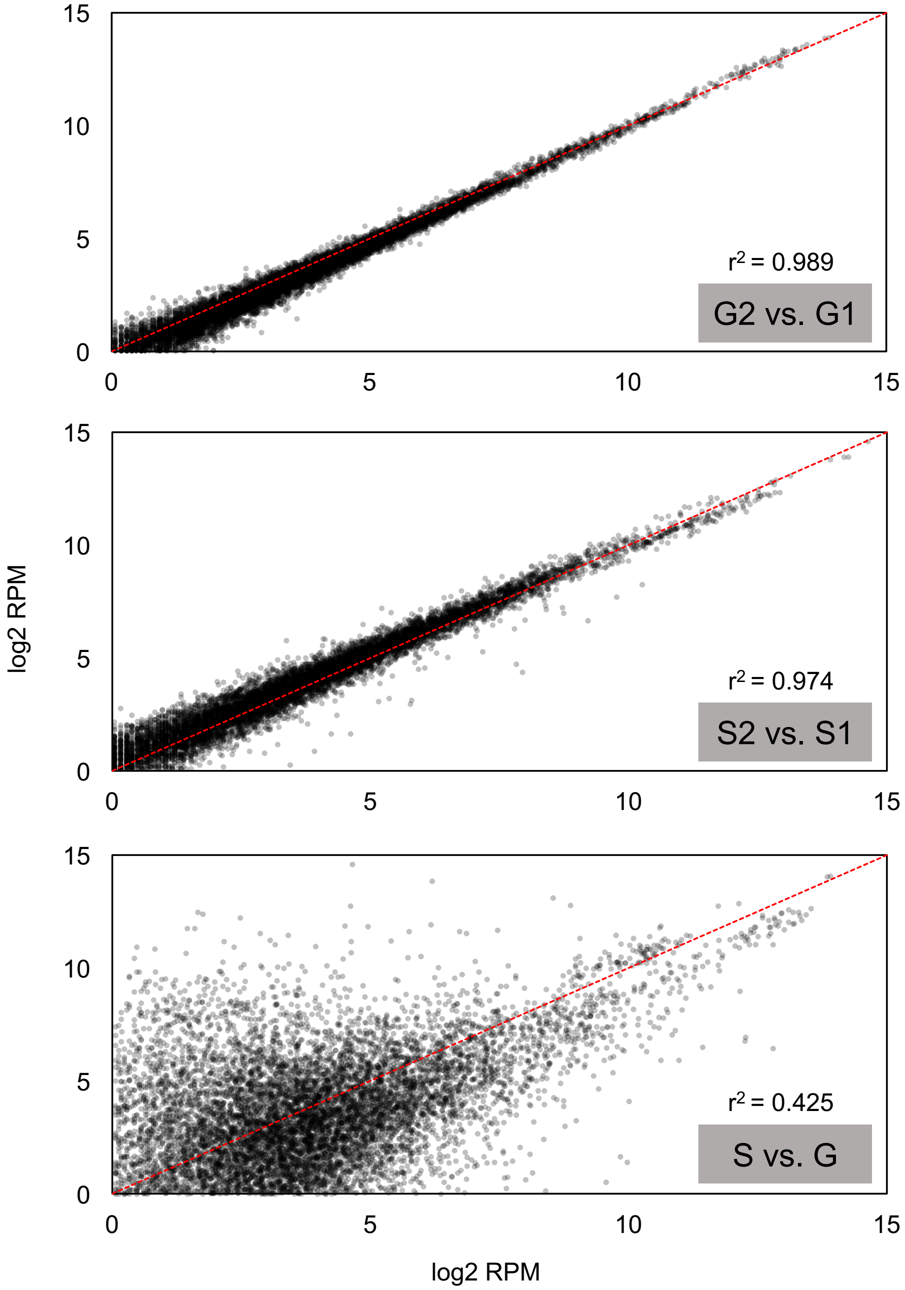

Figure S1. Cell type expression comparisons between replicates and cell types. Correlation of gene expression between gonidial replicate samples (G2 vs. G1), somatic replicate samples (S2 vs. S1), and means for cell type samples (S vs. G) plotted on a log scale. Red dashed line represents perfect correlation (r^2=1).

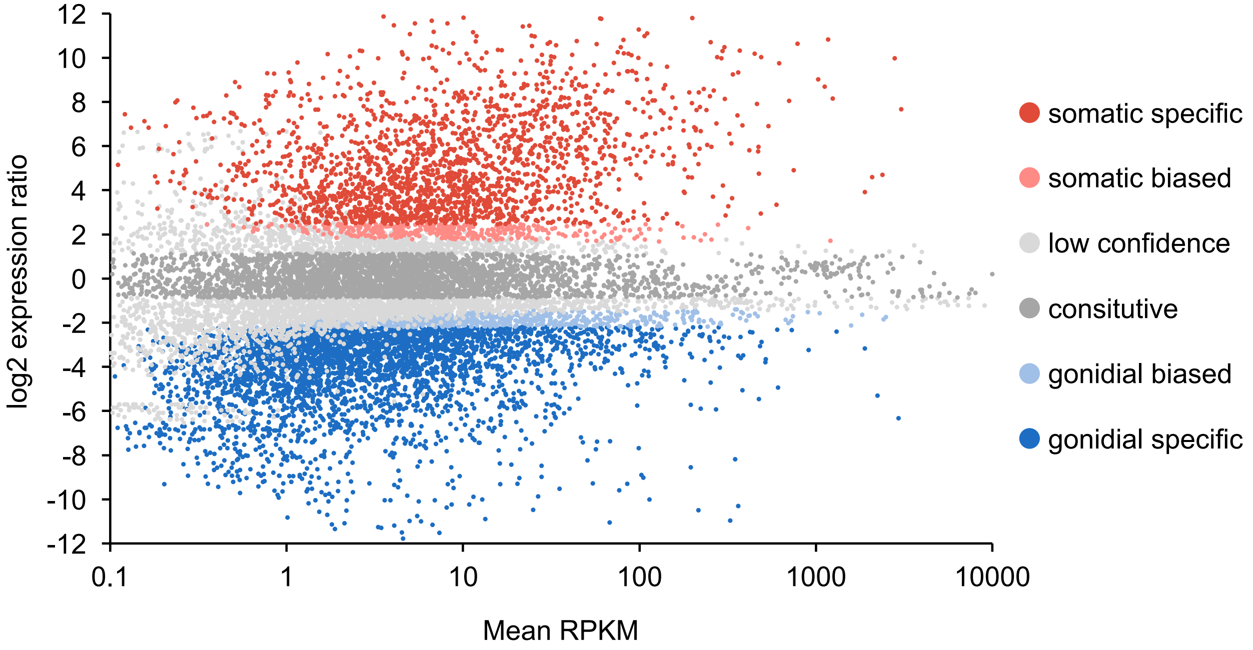

Figure S2. MA plot of the Volvox germ-soma transcriptomes. Each dot represents a single gene with coloring to represent expression category shown in key on right. Mean RPKM computed across cell types is plotted on x-axis, log scale. Somatic/gonidial expression ratio is plotted on y-axis, log2 scale.

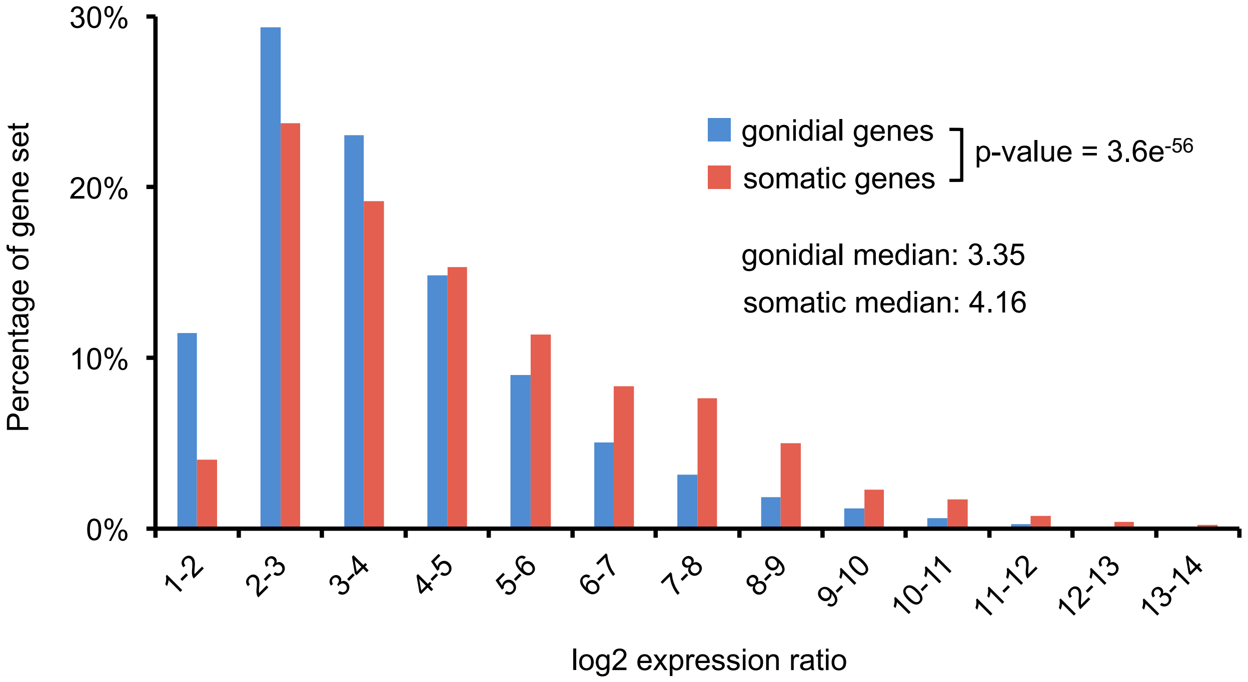

Figure S3. Distributions of expression ratios in the gonidial versus somatic gene sets without flagella genes. Same plot as Figure 3B but with flagella genes removed and significance recalculated.

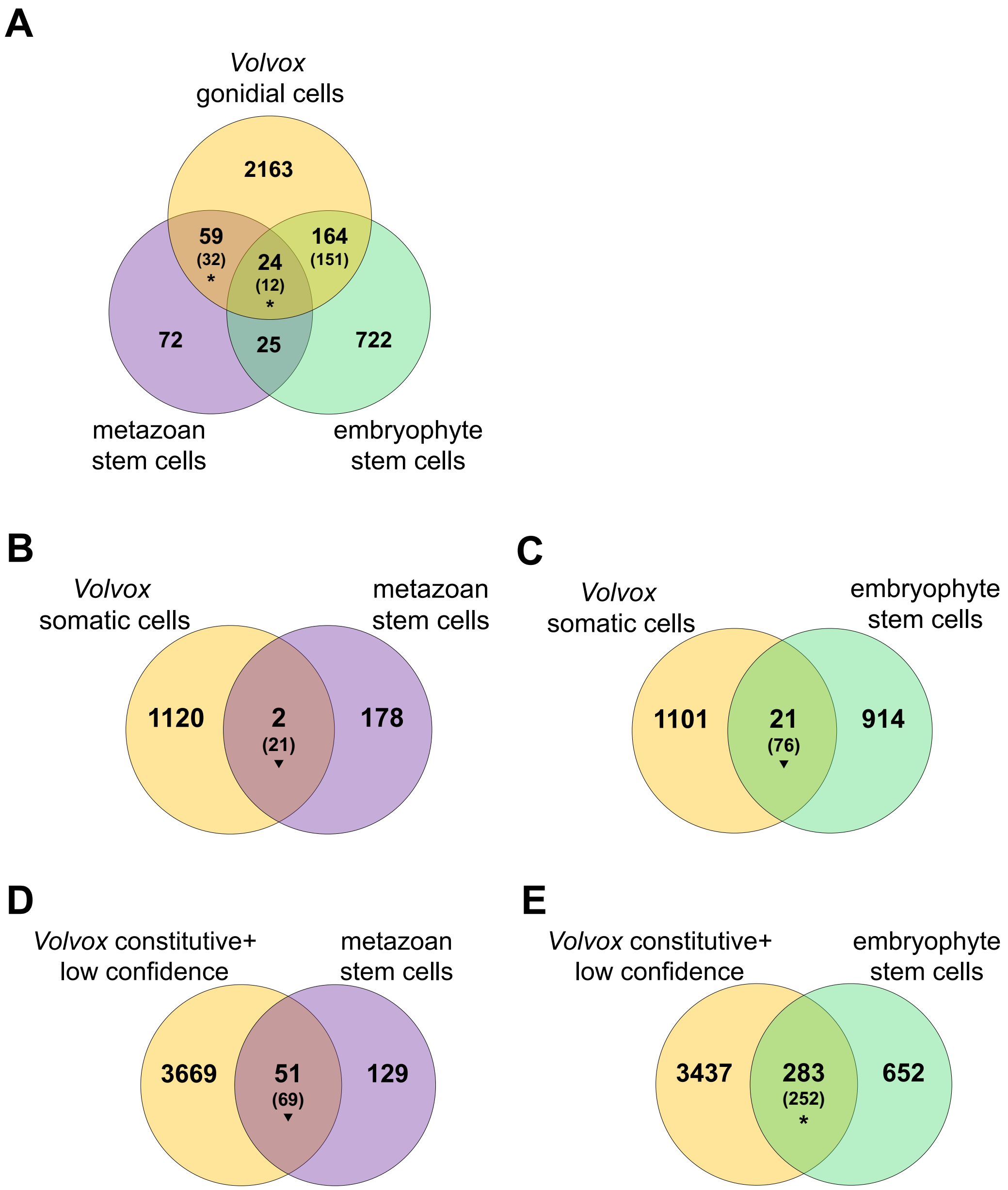

Figure S4. Overlap of OrthoMCL IDs from Volvox cell type genes, metazoan stem cell genes, and embryophyte stem cell genes. (A) Three-way comparison between OrthoMCL IDs associated with Volvox gonidial cells, metazoan stem cells, and embryophyte stem cells. (B-E) Venn diagrams similar to those in Figure 4B,C show overlaps between OrthoMCL IDs associated with either Volvox somatic cells (B,C) or a combined set of Volvox constitutive and low-confidence genes (D,E) and either metazoan stem cells (B,D) or embryophyte stem cells (C,E). Asterisks indicate significant enrichment (* FDR < 0.01) and inverted triangles indicate significant de-enrichment (▼ FDR < 0.01) compared to expected overlap values shown in parentheses.

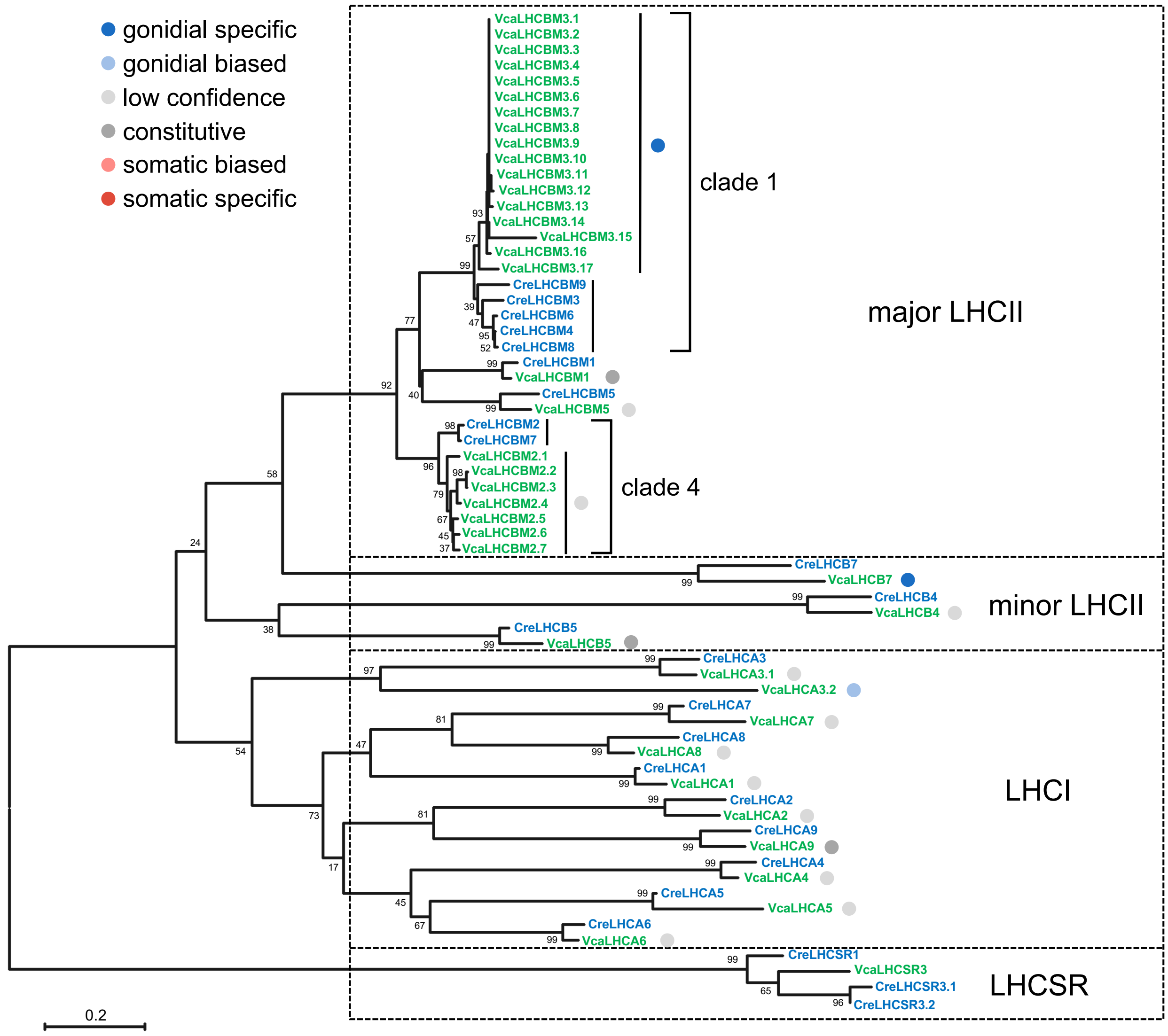

Figure S5. Neighbor joining tree showing Volvox and Chlamydomonas LHC gene family phylogenies with Volvox cell type expression classifications. The tree is drawn to scale, with branch lengths in the same units as those of the evolutionary distances used to infer the phylogenetic tree (scale bar, 0.2 amino acid substitutions per site). Volvox and Chlamydomonas proteins are colored in green and blue, respectively. Bootstrap values (percentages from 1000 replicates) are indicated at nodes. “clade 1” and “clade 4” indicate groups of highly similar paralogs whose Volvox members were collapsed into single clade 1 or clade 4 meta-genes to estimate cell type expression (see Materials and Methods). Colored dots mark expression classifications of genes/meta-genes according to the key at the top left. The expression patterns of genes encoding LHCSR proteins were not included in this analysis

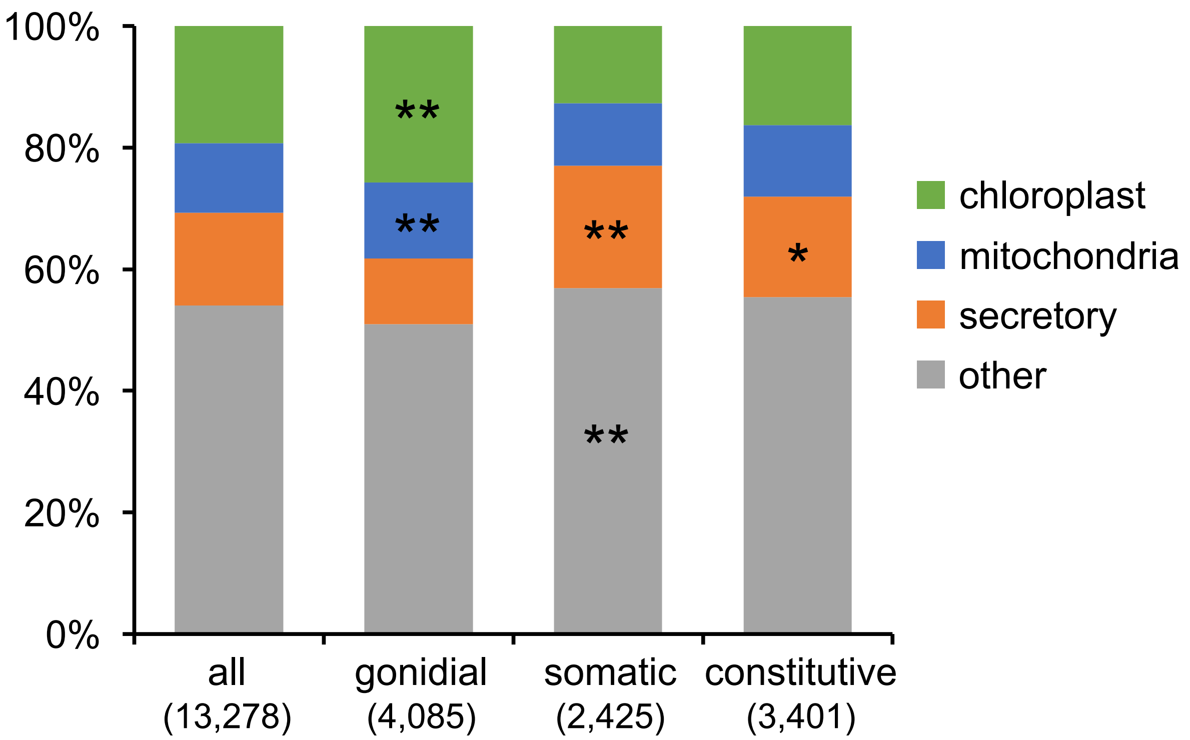

Figure S6. Subcellular localization predictions for Volvox proteins from different expression classifications. Bar graphs showing fractional distributions of subcellular localization predictions of proteins encoded by gonidial, somatic, and constitutively expressed gene sets. Color coding for graph is in the key on the right. Number of predicted proteins in each data set is shown in parentheses below each bar. Asterisks indicate significant enrichment of a localization category as determined by a Fisher's exact test (** FDR < 0.01, * FDR < 0.05).

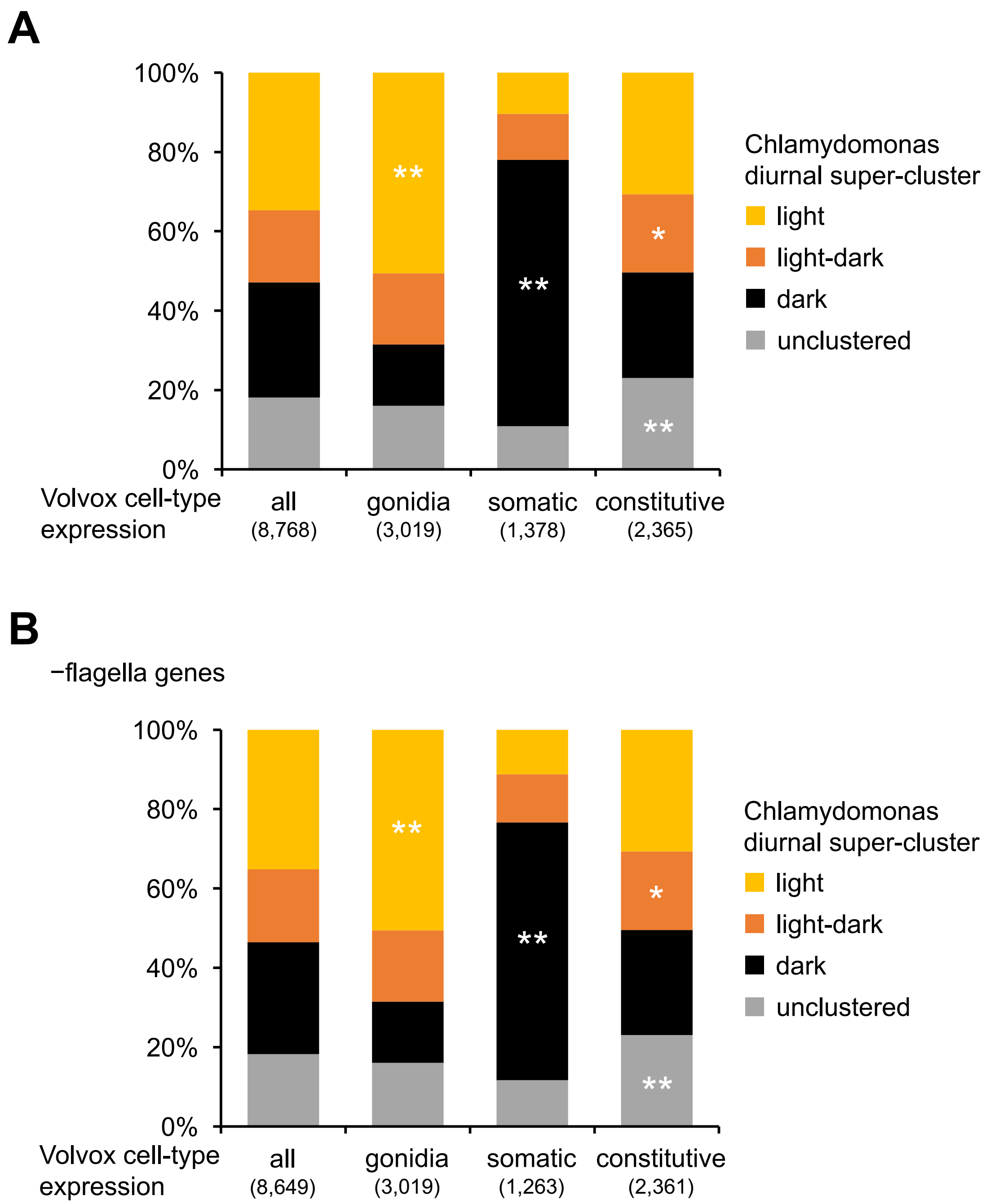

Figure S7. Diurnal super-cluster distributions for Chlamydomonas orthologs of Volvox cell-type genes. (A) Stacked bar plots show fractions of Chlamydomonas genes in each of the designated super-clusters whose Volvox orthologs belonged to the indicated cell type classifications (all genes, gonidial, somatic, constitutive). Asterisks indicate significant enrichment determined using Fisher's exact test (** FDR < 0.01, * FDR < 0.05). (B) Same analysis as in (A) but with flagella genes removed.

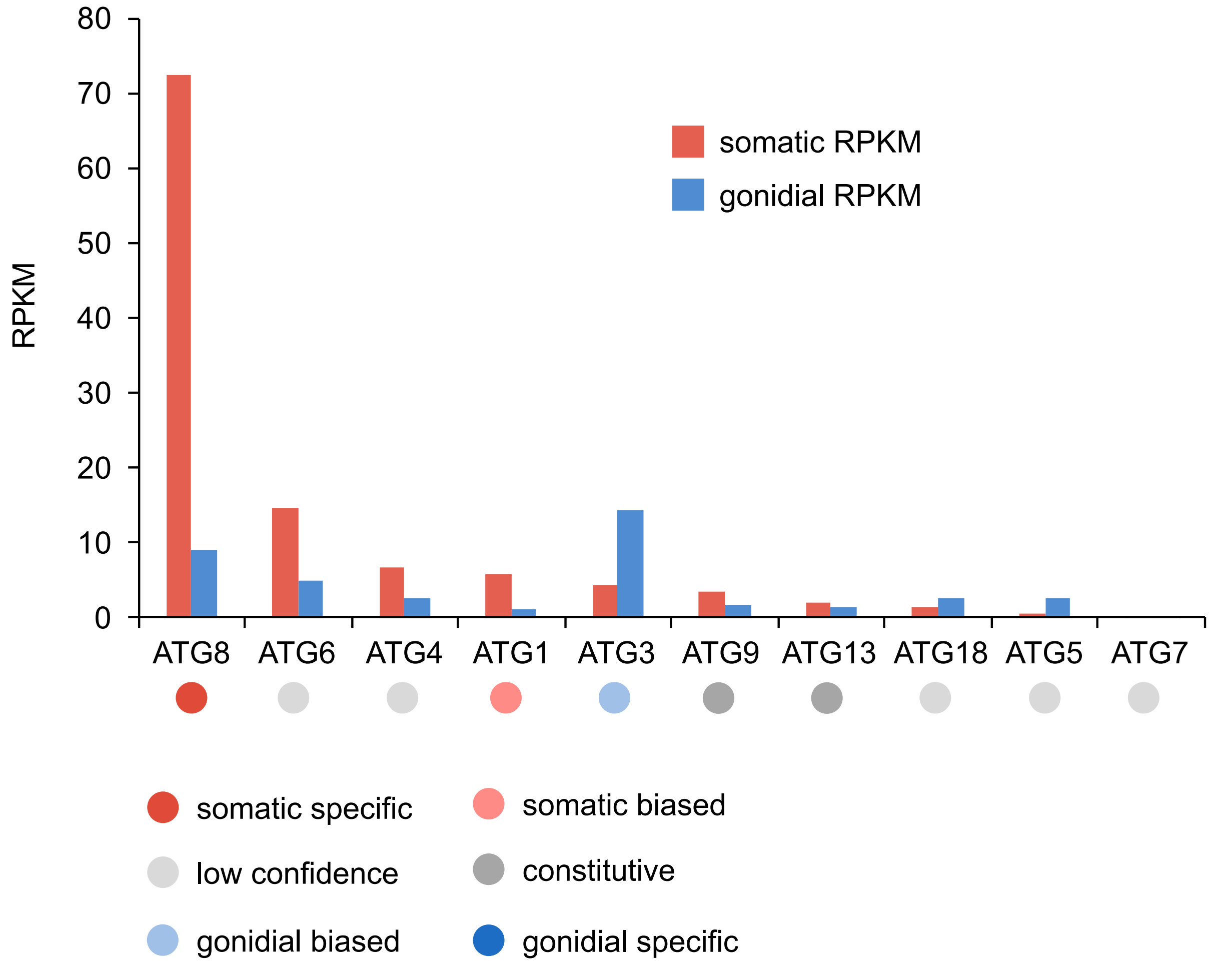

Figure S8. Expression patterns of autophagy-related (ATG) genes. Bar plot shows cell type expression (reads per million per kilobase or RPKM, y axis) of Volvox autophagy-related (ATG) genes (see Dataset S8). Colored dots mark expression classifications of genes according to the key at the bottom.

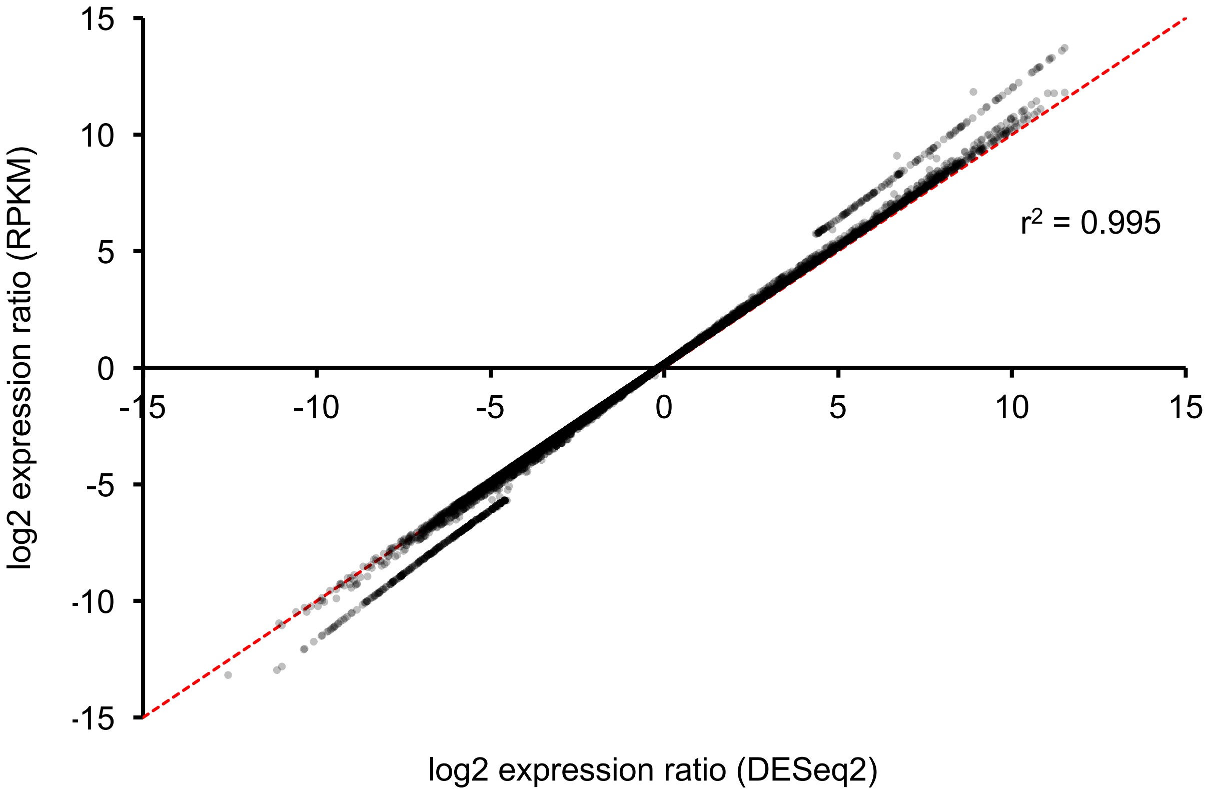

Figure S9. Effect of DESeq2 normalization on average expression values. Correlation of cell-type expression ratios derived from RPKM normalization versus DESeq2 normalization. Positive values indicate somatic-specific expression and negative values indicate gonidial-specific expression. The red dashed line marks perfect correlation (r^2=1).

Table S1. RNA-seq read mapping metrics.

	gonidia1	gonidia2	somatic1	somatic2
Number of reads	15,572,992	15,626,215	16,400,552	17,218,685
Number of high-quality reads	14,901,143	14,965,921	15,584,482	16,471,537
Number of reads uniquely mapping to genome	12,883,915	12,934,061	12,247,559	14,654,399
Percent of reads uniquely mapping to genome	86.46%	86.42%	78.59%	88.97%
Number of reads uniquely mapping to gene models	11,583,555	11,650,101	10,838,222	13,104,583
Percent of reads uniquely mapping to gene models	77.74%	77.84%	69.54%	79.56%

Table S2. Numbers of cell-type genes exhibiting high expression ratios between cell types.

expression ratio	Gonidial genes (G/S>10) (# of genes)	Somatic genes (S/G>10) (# of genes)
>1000	41	81
>100	388	528
>10	2264	1712

Table S3. MapMan terms enriched in proteins encoded by the gonidial, somatic, and constitutive gene sets.

Gonidial enriched terms			
MapMan term	*Observed*	*Expected*	*FDR*
Deoxynucleotide metabolism	10	4	2.14E-02
DNA synthesis/chromatin structure	63	39	1.94E-04
DNA repair	29	16	4.97E-03
RNA processing	74	54	3.27E-02
Amino acid synthesis	42	24	1.24E-03
Arginine biosynthesis	10	4	7.47E-03
Lysine biosynthesis	7	2	1.21E-02
Protein assembly and cofactor ligation	9	4	4.08E-02
Tetrapyrrole synthesis	22	13	2.2E-02
Co-factor and vitamin metabolism	22	14	4.8E-02
Protein targeting	67	48	1.98E-02
Metalloproteases	25	14	3.51E-02
**Somatic enriched terms**			
MapMan term	*Observed*	*Expected*	*FDR*
Cell motility	126	25	2.33E-69
Post-translational modification	107	54	4.06E-11
Cell organization	28	15	2.10E-02
**Constitutive enriched terms**			
MapMan term	*Observed*	*Expected*	*FDR*
Protein synthesis	103	56	8.10E-10
Mitochondrial electron transport / ATP synthesis	44	20	2.2E-07
ATPases	12	5	2.01E-02
Transcriptional regulation	92	68	4.08E-02

Table S4. **GO terms enriched in the proteins encoded by the gonidial, somatic, and constitutive gene sets.**

**Gonidial enriched terms**				
GO-ID	*Term*	*Observed*	*Expected*	*FDR*
GO:0090304	nucleic acid metabolic process	230	163	2.83E-09
GO:0006259	DNA metabolic process	141	98	1.27E-06
GO:0003676	nucleic acid binding	333	277	1.17E-04
GO:0016070	RNA metabolic process	92	67	1.04E-03
GO:0034660	ncRNA metabolic process	67	41	2.28E-05
GO:0006399	tRNA metabolic process	67	41	2.28E-05
GO:0003723	RNA binding	158	118	5.71E-05
GO:0008135	translation factor activity, RNA binding	37	25	1.31E-02
GO:0051301	cell division	30	21	3.71E-02
GO:0006807	nitrogen compound metabolic process	829	744	5.71E-05
GO:0051186	cofactor metabolic process	168	130	2.84E-04
GO:0009536	plastid	346	240	1.45E-15
GO:0006091	generation of precursor metabolites and energy	130	108	2.25E-02
GO:0015979	photosynthesis	86	60	2.98E-04
GO:0009058	biosynthetic process	682	580	6.80E-08
GO:0006629	lipid metabolic process	172	140	2.98E-03
GO:0006886	intracellular protein transport	63	40	1.34E-04
GO:0033036	macromolecule localization	63	40	1.34E-04
GO:0005739	mitochondrion	118	93	6.52E-03
GO:0016482	cytoplasmic transport	30	20	2.33E-02
GO:0008168	methyltransferase activity	104	65	1.55E-07
**Somatic enriched terms**				
GO-ID	*Term*	*Observed*	*Expected*	*FDR*
GO:0005929	cilium	202	41	5.51E-117

GO:0005856	cytoskeleton	100	32	3.88E-28
GO:0007165	signal transduction	131	62	1.38E-17
GO:0007154	cell communication	131	63	2.58E-17
GO:0000902	cell morphogenesis	43	16	4.77E-10
GO:0030705	cytoskeleton-dependent intracellular transport	12	2	2.23E-08
GO:0015630	microtubule cytoskeleton	20	6	7.65E-07
GO:0036211	protein modification process	220	187	9.41E-03
GO:0016798	hydrolase activity, acting on glycosyl bonds	22	13	2.64E-02
**Constitutive enriched terms**				
GO-ID	*Term*	*Observed*	*Expected*	*FDR*
GO:0006412	translation	115	64	1.54E-11
GO:0010467	gene expression	151	92	3.14E-11
GO:0042393	histone binding	7	3	3.00E-02
GO:0042254	ribosome biogenesis	126	85	4.56E-06
GO:0012505	endomembrane system	89	63	1.65E-03
GO:0006091	generation of precursor metabolites and energy	115	89	1.05E-02
GO:0036211	protein modification process	345	303	1.42E-02
GO:0005794	Golgi apparatus	43	29	1.73E-02
GO:0005783	endoplasmic reticulum	44	30	2.78E-02
GO:0005739	mitochondrion	97	77	3.60E-02

Supplemental tables and datasets (excel format):

Table S5. Proteomes used for phylostratigraphy.

Dataset S1. Expression of all genes.

Dataset S2. Expression of VARL genes.

Dataset S3. Expression of genes with previously published cell-type expression data.

Dataset S4. Expression of flagella, basal body, and transition zone genes.

Dataset S5. Expression of photosynthesis genes.

Dataset S6. Expression of carbon metabolism genes.

Dataset S7. Expression of nucleotide sugar metabolism genes.

Dataset S8. Expression of glycosyltransferase genes.

Dataset S9. Expression of extracellular matrix (ECM) genes.

Dataset S10. Expression of autophagy genes.

Supplemental references

1. Hanschen ER, Marriage TN, Ferris PJ, Hamaji T, Toyoda A, Fujiyama A, et al. The Gonium pectorale genome demonstrates co-option of cell cycle regulation during the evolution of multicellularity. Nat Commun. 2016;7: 11370. doi:10.1038/ncomms11370
2. Merchant SS, Prochnik SE, Vallon O, Harris EH, Karpowicz SJ, Witman GB, et al. The Chlamydomonas genome reveals the evolution of key animal and plant functions. Science. 2007;318: 245–50. doi:10.1126/science.1143609
3. Blanc G, Duncan G, Agarkova I, Borodovsky M, Gurnon J, Kuo A, et al. The Chlorella variabilis NC64A genome reveals adaptation to photosymbiosis, coevolution with viruses, and cryptic sex. Plant Cell. 2010;22: 2943–55. doi:10.1105/tpc.110.076406
4. Blanc G, Agarkova I, Grimwood J, Kuo A, Brueggeman A, Dunigan DD, et al. The genome of the polar eukaryotic microalga Coccomyxa subellipsoidea reveals traits of cold adaptation. Genome Biol. 2012;13: R39. doi:10.1186/gb-2012-13-5-r39
5. Palenik B, Grimwood J, Aerts A, Rouzé P, Salamov A, Putnam N, et al. The tiny eukaryote Ostreococcus provides genomic insights into the paradox of plankton speciation. Proc Natl Acad Sci U S A. 2007;104: 7705–10. doi:10.1073/pnas.0611046104
6. Worden AZ, Lee J-H, Mock T, Rouzé P, Simmons MP, Aerts AL, et al. Green evolution and dynamic adaptations revealed by genomes of the marine picoeukaryotes Micromonas. Science. 2009;324: 268–72. doi:10.1126/science.1167222
7. Hori K, Maruyama F, Fujisawa T, Togashi T, Yamamoto N, Seo M, et al. Klebsormidium flaccidum genome reveals primary factors for plant terrestrial adaptation. Nat Commun. Nature Publishing Group; 2014;5: 3978. doi:10.1038/ncomms4978
8. Matsuzaki M, Misumi O, Shin-I T, Maruyama S, Takahara M, Miyagishima S-Y, et al. Genome sequence of the ultrasmall unicellular red alga Cyanidioschyzon merolae 10D. Nature. 2004;428: 653–7. doi:10.1038/nature02398
9. Lamesch P, Berardini TZ, Li D, Swarbreck D, Wilks C, Sasidharan R, et al. The Arabidopsis Information Resource (TAIR): improved gene annotation and new tools. Nucleic Acids Res. 2012;40: D1202-10. doi:10.1093/nar/gkr1090
10. Ouyang S, Zhu W, Hamilton J, Lin H, Campbell M, Childs K, et al. The TIGR Rice Genome Annotation Resource: improvements and new features. Nucleic Acids Res. 2007;35: D883-7. doi:10.1093/nar/gkl976
11. Rensing SA, Lang D, Zimmer AD, Terry A, Salamov A, Shapiro H, et al. The Physcomitrella genome reveals evolutionary insights into the conquest of land by plants. Science. 2008;319: 64–9. doi:10.1126/science.1150646
12. C. elegans Sequencing Consortium. Genome sequence of the nematode C. elegans: a platform for investigating biology. Science. 1998;282: 2012–8.
13. Adams MD, Celniker SE, Holt RA, Evans CA, Gocayne JD, Amanatides PG, et al. The genome sequence of Drosophila melanogaster. Science. 2000;287: 2185–95.
14. Colbourne JK, Pfrender ME, Gilbert D, Thomas WK, Tucker A, Oakley TH, et al.

The ecoresponsive genome of Daphnia pulex. Science. 2011;331: 555–61. doi:10.1126/science.1197761
15. Lander ES, Linton LM, Birren B, Nusbaum C, Zody MC, Baldwin J, et al. Initial sequencing and analysis of the human genome. Nature. 2001;409: 860–921. doi:10.1038/35057062
16. King N, Westbrook MJ, Young SL, Kuo A, Abedin M, Chapman J, et al. The genome of the choanoflagellate Monosiga brevicollis and the origin of metazoans. Nature. 2008;451: 783–8. doi:10.1038/nature06617
17. Cherry JM, Hong EL, Amundsen C, Balakrishnan R, Binkley G, Chan ET, et al. Saccharomyces Genome Database: the genomics resource of budding yeast. Nucleic Acids Res. 2012;40: D700-5. doi:10.1093/nar/gkr1029
18. Galagan JE, Calvo SE, Borkovich KA, Selker EU, Read ND, Jaffe D, et al. The genome sequence of the filamentous fungus Neurospora crassa. Nature. 2003;422: 859–68. doi:10.1038/nature01554
19. Eichinger L, Pachebat JA, Glöckner G, Rajandream M-A, Sucgang R, Berriman M, et al. The genome of the social amoeba Dictyostelium discoideum. Nature. 2005;435: 43–57. doi:10.1038/nature03481
20. Clarke M, Lohan AJ, Liu B, Lagkouvardos I, Roy S, Zafar N, et al. Genome of Acanthamoeba castellanii highlights extensive lateral gene transfer and early evolution of tyrosine kinase signaling. Genome Biol. 2013;14: R11. doi:10.1186/gb-2013-14-2-r11
21. Bowler C, Allen AE, Badger JH, Grimwood J, Jabbari K, Kuo A, et al. The Phaeodactylum genome reveals the evolutionary history of diatom genomes. Nature. 2008;456: 239–44. doi:10.1038/nature07410
22. Cock JM, Sterck L, Rouzé P, Scornet D, Allen AE, Amoutzias G, et al. The Ectocarpus genome and the independent evolution of multicellularity in brown algae. Nature. 2010;465: 617–21. doi:10.1038/nature09016
23. Corteggiani Carpinelli E, Telatin A, Vitulo N, Forcato C, D'Angelo M, Schiavon R, et al. Chromosome scale genome assembly and transcriptome profiling of Nannochloropsis gaditana in nitrogen depletion. Mol Plant. 2014;7: 323–35. doi:10.1093/mp/sst120
24. Eisen JA, Coyne RS, Wu M, Wu D, Thiagarajan M, Wortman JR, et al. Macronuclear genome sequence of the ciliate Tetrahymena thermophila, a model eukaryote. PLoS Biol. 2006;4: e286. doi:10.1371/journal.pbio.0040286
25. Stover NA, Krieger CJ, Binkley G, Dong Q, Fisk DG, Nash R, et al. Tetrahymena Genome Database (TGD): a new genomic resource for Tetrahymena thermophila research. Nucleic Acids Res. 2006;34: D500-3. doi:10.1093/nar/gkj054
26. Gardner MJ, Hall N, Fung E, White O, Berriman M, Hyman RW, et al. Genome sequence of the human malaria parasite Plasmodium falciparum. Nature. 2002;419: 498–511. doi:10.1038/nature01097
27. Blattner FR, Plunkett G, Bloch CA, Perna NT, Burland V, Riley M, et al. The complete genome sequence of Escherichia coli K-12. Science. 1997;277: 1453–62.
28. Glasner JD, Liss P, Plunkett G, Darling A, Prasad T, Rusch M, et al. ASAP, a systematic annotation package for community analysis of genomes. Nucleic Acids Res. 2003;31: 147–51.

29. Kunst F, Ogasawara N, Moszer I, Albertini AM, Alloni G, Azevedo V, et al. The complete genome sequence of the gram-positive bacterium Bacillus subtilis. Nature. 1997;390: 249–56. doi:10.1038/36786
30. Goldman BS, Nierman WC, Kaiser D, Slater SC, Durkin a S, Eisen J a, et al. Evolution of sensory complexity recorded in a myxobacterial genome. Proc Natl Acad Sci U S A. 2006;103: 15200–5. doi:10.1073/pnas.0607335103
31. Dufresne A, Ostrowski M, Scanlan DJ, Garczarek L, Mazard S, Palenik BP, et al. Unraveling the genomic mosaic of a ubiquitous genus of marine cyanobacteria. Genome Biol. 2008;9: R90. doi:10.1186/gb-2008-9-5-r90
32. Shih PM, Wu D, Latifi A, Axen SD, Fewer DP, Talla E, et al. Improving the coverage of the cyanobacterial phylum using diversity-driven genome sequencing. Proc Natl Acad Sci U S A. 2013;110: 1053–8. doi:10.1073/pnas.1217107110
33. Thiel T, Pratte BS, Zhong J, Goodwin L, Copeland A, Lucas S, et al. Complete genome sequence of Anabaena variabilis ATCC 29413. Stand Genomic Sci. 2014;9: 562–73. doi:10.4056/sigs.3899418
34. Klenk HP, Clayton RA, Tomb JF, White O, Nelson KE, Ketchum KA, et al. The complete genome sequence of the hyperthermophilic, sulphate-reducing archaeon Archaeoglobus fulgidus. Nature. 1997;390: 364–70. doi:10.1038/37052
35. Bult CJ, White O, Olsen GJ, Zhou L, Fleischmann RD, Sutton GG, et al. Complete genome sequence of the methanogenic archaeon, Methanococcus jannaschii. Science. 1996;273: 1058–73.
36. Pfeiffer F, Schuster SC, Broicher A, Falb M, Palm P, Rodewald K, et al. Evolution in the laboratory: the genome of Halobacterium salinarum strain R1 compared to that of strain NRC-1. Genomics. 2008;91: 335–46. doi:10.1016/j.ygeno.2008.01.001
37. Fitz-Gibbon ST, Ladner H, Kim U-J, Stetter KO, Simon MI, Miller JH. Genome sequence of the hyperthermophilic crenarchaeon Pyrobaculum aerophilum. Proc Natl Acad Sci U S A. 2002;99: 984–9. doi:10.1073/pnas.241636498
38. Kawarabayasi Y, Hino Y, Horikawa H, Yamazaki S, Haikawa Y, Jin-no K, et al. Complete genome sequence of an aerobic hyper-thermophilic crenarchaeon, Aeropyrum pernix K1. DNA Res. 1999;6: 83–101, 145–52.
39. She Q, Singh RK, Confalonieri F, Zivanovic Y, Allard G, Awayez MJ, et al. The complete genome of the crenarchaeon Sulfolobus solfataricus P2. Proc Natl Acad Sci U S A. 2001;98: 7835–40. doi:10.1073/pnas.141222098
40. Zones JM, Blaby IK, Merchant SS, Umen JG. High-Resolution Profiling of a Synchronized Diurnal Transcriptome from Chlamydomonas reinhardtii Reveals Continuous Cell and Metabolic Differentiation. Plant Cell. 2015;27: 2743–2769. doi:10.1105/tpc.15.00498
41. Barker AR, Renzaglia KS, Fry K, Dawe HR. Bioinformatic analysis of ciliary transition zone proteins reveals insights into the evolution of ciliopathy networks. BMC Genomics. 2014;15: 531. doi:10.1186/1471-2164-15-531
42. Hodges ME, Scheumann N, Wickstead B, Langdale JA, Gull K. Reconstructing the evolutionary history of the centriole from protein components. J Cell Sci. 2010;123: 1407–13. doi:10.1242/jcs.064873
43. Feldman JL, Marshall WF. ASQ2 Encodes a TBCC-like Protein Required for

Mother-Daughter Centriole Linkage and Mitotic Spindle Orientation. Curr Biol. Elsevier Ltd; 2009;19: 1238–1243. doi:10.1016/j.cub.2009.05.071
44. Keller LC, Geimer S, Romijn E, Yates J, Zamora I, Marshall WF. Molecular architecture of the centriole proteome: the conserved WD40 domain protein POC1 is required for centriole duplication and length control. Mol Biol Cell. 2009;20: 1150–66. doi:10.1091/mbc.E08-06-0619
45. Elrad D, Grossman AR. A genome's eye view of the light-harvesting polypeptides of Chlamydomonas reinhardtii. Curr Genet. 2004;45: 61–75. doi:10.1007/s00294-003-0460-x
46. Nickelsen J, Rengstl B. Photosystem II assembly: from cyanobacteria to plants. Annu Rev Plant Biol. 2013;64: 609–35. doi:10.1146/annurev-arplant-050312-120124
47. Schöttler MA, Tóth SZ, Boulouis A, Kahlau S. Photosynthetic complex stoichiometry dynamics in higher plants: biogenesis, function, and turnover of ATP synthase and the cytochrome b6f complex. J Exp Bot. 2015;66: 2373–400. doi:10.1093/jxb/eru495
48. Yang H, Liu J, Wen X, Lu C. Molecular mechanism of photosystem I assembly in oxygenic organisms. Biochim Biophys Acta. 2015;1847: 838–848. doi:10.1016/j.bbabio.2014.12.011
49. Goodenough U, Blaby I, Casero D, Gallaher SD, Goodson C, Johnson S, et al. The path to triacylglyceride obesity in the sta6 strain of Chlamydomonas reinhardtii. Eukaryot Cell. 2014;13: 591–613. doi:10.1128/EC.00013-14
50. Ball SG, Deschamps P. Chapter 1- Starch Metabolism. In: Stern DB, Harris EH, editors. The Chlamydomonas Sourcebook Vol 2. 2nd ed. San Diego, CA: Academic Press, Inc; 2009.
51. Hemschemeier A, Casero D, Liu B, Benning C, Pellegrini M, Happe T, et al. Copper response regulator1-dependent and -independent responses of the Chlamydomonas reinhardtii transcriptome to dark anoxia. Plant Cell. 2013;25: 3186–3211. doi:10.1105/tpc.113.115741
52. Gargouri M, Park JJ, Holguin FO, Kim MJ, Wang H, Deshpande RR, et al. Identification of regulatory network hubs that control lipid metabolism in Chlamydomonas reinhardtii. J Exp Bot. 2015;66: 4551–4566. doi:10.1093/jxb/erv217
53. Blaby IK, Blaby-Haas CE, Pérez-Pérez ME, Schmollinger S, Fitz-Gibbon S, Lemaire SD, et al. Genome-wide analysis on Chlamydomonas reinhardtii reveals the impact of hydrogen peroxide on protein stress responses and overlap with other stress transcriptomes. Plant J. 2015;84: 974–988. doi:10.1111/tpj.13053
54. Burgess SJ, Taha H, Yeoman JA, Iamshanova O, Chan KX, Boehm M, et al. Identification of the Elusive Pyruvate Reductase of Chlamydomonas reinhardtii Chloroplasts. Plant Cell Physiol. 2016;57: 82–94. doi:10.1093/pcp/pcv167
55. Li-Beisson Y, Beisson F, Riekhof W. Metabolism of acyl-lipids in Chlamydomonas reinhardtii. Plant J. 2015;82: 504–522. doi:10.1111/tpj.12787
56. Knoop H, Gründel M, Zilliges Y, Lehmann R, Hoffmann S, Lockau W, et al. Flux Balance Analysis of Cyanobacterial Metabolism: The Metabolic Network of Synechocystis sp. PCC 6803. PLoS Comput Biol. 2013;9. doi:10.1371/journal.pcbi.1003081

57. Vallon O, Spalding MH. Chapter 4 - Amino Acid Metabolism. In: Stern DB, Harris EH, editors. The Chlamydomonas Sourcebook Vol 2. 2nd ed. San Diego, CA: Academic Press, Inc; 2009.
58. Fernandez E, Llamas A, Galvan A. Chapter 3 - Nitrogen Assimilation and its Regulation. In: Stern DB, Harris EH, editors. The Chlamydomonas Sourcebook Vol 2. 2nd ed. San Diego, CA: Academic Press, Inc; 2009.
59. Fontaine J-X, Terce-Laforgue T, Armengaud P, Clement G, Renou J-P, Pelletier S, et al. Characterization of a NADH-Dependent Glutamate Dehydrogenase Mutant of Arabidopsis Demonstrates the Key Role of this Enzyme in Root Carbon and Nitrogen Metabolism. Plant Cell. 2012;24: 4044–4065. doi:10.1105/tpc.112.103689
60. Prochnik SE, Umen J, Nedelcu AM, Hallmann A, Miller SM, Nishii I, et al. Genomic analysis of organismal complexity in the multicellular green alga Volvox carteri. Science. 2010;329: 223–6. doi:10.1126/science.1188800
61. Shimizu T, Inoue T, Shiraishi H. Cloning and characterization of novel extensin-like cDNAs that are expressed during late somatic cell phase in the green alga Volvox carteri. Gene. 2002;284: 179–187.
62. Amon P, Haas E, Sumper M. The Sex-Inducing Pheromone and Wounding Trigger the Same Set of Genes in the Multicellular Green Alga Volvox. Plant Cell. 1998;10: 781–789. doi:10.1105/tpc.10.5.781
63. Nishimura M, Nagashio R, Sato Y, Hasegawa T. Late Somatic Gene 2 disrupts parental spheroids cooperatively with Volvox hatching enzyme A in Volvox. Planta. 2016; doi:10.1007/s00425-016-2599-y
64. Hallmann A. Enzymes in the Extracellular Matrix of Volvox: an Inducible, Calcium-dependent Phosphatase with a Modular Composition. J Biol Chem. 1999;274: 1691–1697. doi:10.1074/jbc.274.3.1691
65. Huber O, Sumper M. Algal-CAMs: isoforms of a cell adhesion molecule in embryos of the alga Volvox with homology to Drosophila fasciclin I. EMBO J. 1994;13: 4212–22.
66. Hallmann A, Sumper M. An inducible arylsulfatase of Volvox carteri with properties suitable for a reporter-gene system: Purification, characterization and molecular cloning. Eur J Biochem. 1994;221: 143–150. doi:10.1111/j.1432-1033.1994.tb18723.x
67. Klein U. Compartmentation of glycolysis and of the oxidative pentose-phosphate pathway in Chlamydomonas reinhardii. Planta. 1986;167: 81–86. doi:10.1007/BF00446372
68. Johnson X, Alric J. Central carbon metabolism and electron transport in Chlamydomonas reinhardtii: metabolic constraints for carbon partitioning between oil and starch. Eukaryot Cell. 2013;12: 776–93. doi:10.1128/EC.00318-12
69. Seifert GJ. Nucleotide sugar interconversions and cell wall biosynthesis: How to bring the inside to the outside. Curr Opin Plant Biol. 2004;7: 277–284. doi:10.1016/j.pbi.2004.03.004
70. Bar-Peled M, O´Neill MA. Plant Nucleotide Sugar Formation , Interconversion , and Salvage by Sugar. Annu Rev Plant Biol. 2011;67: 127–155. doi:10.1146/annurev-arplant-042110-103918
71. Dauvillée D, Chochois V, Steup M, Haebel S, Eckermann N, Ritte G, et al.

Plastidial phosphorylase is required for normal starch synthesis in Chlamydomonas reinhardtii. Plant J. 2006;48: 274–285. doi:10.1111/j.1365-313X.2006.02870.x
